# Accuracy of Tau Positron Emission Tomography as a Prognostic Marker in Preclinical and Prodromal Alzheimer Disease: A Head-to-Head Comparison Against Amyloid Positron Emission Tomography and Magnetic Resonance Imaging

**DOI:** 10.1001/jamaneurol.2021.1858

**Published:** 2021-06-28

**Authors:** Rik Ossenkoppele, Ruben Smith, Niklas Mattsson-Carlgren, Colin Groot, Antoine Leuzy, Olof Strandberg, Sebastian Palmqvist, Tomas Olsson, Jonas Jögi, Erik Stormrud, Hanna Cho, Young Hoon Ryu, Jae Yong Choi, Adam L. Boxer, Maria L. Gorno-Tempini, Bruce L. Miller, David Soleimani-Meigooni, Leonardo Iaccarino, Renaud La Joie, Suzanne Baker, Edilio Borroni, Gregory Klein, Michael J. Pontecorvo, Michael D. Devous, William J. Jagust, Chul Hyoung Lyoo, Gil D. Rabinovici, Oskar Hansson

**Affiliations:** 1 Clinical Memory Research Unit, Lund University, Malmö, Sweden; 2 Alzheimer Center Amsterdam, Department of Neurology, Amsterdam Neuroscience, Vrije Universiteit Amsterdam, Amsterdam University Medical Center, Amsterdam, the Netherlands; 3 Department of Neurology, Skåne University Hospital, Lund, Sweden; 4 Wallenberg Centre for Molecular Medicine, Lund University, Lund, Sweden; 5 Department of Radiation Physics, Skåne University Hospital, Lund, Sweden; 6 Department of Clinical Physiology and Nuclear Medicine, Skåne University Hospital, Lund, Sweden; 7 Memory Clinic, Skåne University Hospital, Malmö, Sweden; 8 Department of Neurology, Gangnam Severance Hospital, Yonsei University College of Medicine, Seoul, South Korea; 9 Department of Nuclear Medicine, Gangnam Severance Hospital, Yonsei University College of Medicine, Seoul, South Korea; 10 Division of Applied Radiological Imaging, Korea Institute Radiological and Medical Sciences, Seoul, South Korea; 11 Department of Neurology, Memory and Aging Center, University of California, San Francisco; 12 Lawrence Berkeley National Laboratory, Berkeley, California; 13 F. Hoffmann–La Roche Ltd, Basel, Switzerland; 14 Avid Radiopharmaceuticals, Philadelphia, Pennsylvania; 15 Helen Wills Neuroscience Institute, University of California, Berkeley; 16 Department of Radiology and Biomedical Imaging, University of California, San Francisco; 17 Molecular Biophysics and Integrated Bioimaging Division, Lawrence Berkeley National Laboratory, Berkeley, California; 18 Associate Editor, *JAMA Neurology*

## Abstract

**Importance:**

Tau positron emission tomography (PET) tracers have proven useful for the differential diagnosis of dementia, but their utility for predicting cognitive change is unclear.

**Objective:**

To examine the prognostic accuracy of baseline fluorine 18 (^18^F)–flortaucipir and [^18^F]RO948 (tau) PET in individuals across the Alzheimer disease (AD) clinical spectrum and to perform a head-to-head comparison against established magnetic resonance imaging (MRI) and amyloid PET markers.

**Design, Setting, and Participants:**

This prognostic study collected data from 8 cohorts in South Korea, Sweden, and the US from June 1, 2014, to February 28, 2021, with a mean (SD) follow-up of 1.9 (0.8) years. A total of 1431 participants were recruited from memory clinics, clinical trials, or cohort studies; 673 were cognitively unimpaired (CU group; 253 [37.6%] positive for amyloid-β [Aβ]), 443 had mild cognitive impairment (MCI group; 271 [61.2%] positive for Aβ), and 315 had a clinical diagnosis of AD dementia (315 [100%] positive for Aβ).

**Exposures:**

[^18^F]Flortaucipir PET in the discovery cohort (n = 1135) or [^18^F]RO948 PET in the replication cohort (n = 296), T1-weighted MRI (n = 1431), and amyloid PET (n = 1329) at baseline and repeated Mini-Mental State Examination (MMSE) evaluation.

**Main Outcomes and Measures:**

Baseline [^18^F]flortaucipir/[^18^F]RO948 PET retention within a temporal region of interest, MRI-based AD-signature cortical thickness, and amyloid PET Centiloids were used to predict changes in MMSE using linear mixed-effects models adjusted for age, sex, education, and cohort. Mediation/interaction analyses tested whether associations between baseline tau PET and cognitive change were mediated by baseline MRI measures and whether age, sex, and *APOE* genotype modified these associations.

**Results:**

Among 1431 participants, the mean (SD) age was 71.2 (8.8) years; 751 (52.5%) were male. Findings for [^18^F]flortaucipir PET predicted longitudinal changes in MMSE, and effect sizes were stronger than for AD-signature cortical thickness and amyloid PET across all participants (*R*^2^, 0.35 [tau PET] vs 0.24 [MRI] vs 0.17 [amyloid PET]; *P* < .001, bootstrapped for difference) in the Aβ-positive MCI group (*R*^2^, 0.25 [tau PET] vs 0.15 [MRI] vs 0.07 [amyloid PET]; *P* < .001, bootstrapped for difference) and in the Aβ-positive CU group (*R*^2^, 0.16 [tau PET] vs 0.08 [MRI] vs 0.08 [amyloid PET]; *P* < .001, bootstrapped for difference). These findings were replicated in the [^18^F]RO948 PET cohort. MRI mediated the association between [^18^F]flortaucipir PET and MMSE in the groups with AD dementia (33.4% [95% CI, 15.5%-60.0%] of the total effect) and Aβ-positive MCI (13.6% [95% CI, 0.0%-28.0%] of the total effect), but not the Aβ-positive CU group (3.7% [95% CI, −17.5% to 39.0%]; *P* = .71). Age (*t* = −2.28; *P* = .02), but not sex (*t* = 0.92; *P* = .36) or *APOE* genotype (*t* = 1.06; *P* = .29) modified the association between baseline [^18^F]flortaucipir PET and cognitive change, such that older individuals showed faster cognitive decline at similar tau PET levels.

**Conclusions and Relevance:**

The findings of this prognostic study suggest that tau PET is a promising tool for predicting cognitive change that is superior to amyloid PET and MRI and may support the prognostic process in preclinical and prodromal stages of AD.

## Key Points

**Question:**

What is the prognostic value of tau positron emission tomography (PET) for predicting cognitive decline across the clinical spectrum of Alzheimer disease?

**Findings:**

In this longitudinal, multicenter prognostic study including 1431 participants, baseline tau PET predicted change in Mini-Mental State Examination scores during a mean (SD) follow-up of 1.9 (0.8) years. Moreover, tau PET outperformed established volumetric magnetic resonance imaging and amyloid PET markers in head-to-head comparisons, especially in participants with mild cognitive impairment and cognitively normal individuals who were positive for amyloid-β.

**Meaning:**

These findings suggest that tau PET is a promising prognostic tool for predicting cognitive decline in preclinical and prodromal stages of Alzheimer disease.

## Teaser

This prognostic study examines the prognostic accuracy of baseline [^18^F]flortaucipir and [^18^F]RO948 (tau) positron emission tomography (PET) in individuals across the Alzheimer disease clinical spectrum and performs a head-to-head comparison with established magnetic resonance imaging and amyloid PET markers.

## Introduction

An accurate prognosis for individuals with Alzheimer disease (AD) is essential for patients and families to plan for the future, reduce uncertainty, increase safety, and optimize medical decision-making.^1^ Despite the development of several biomarkers for neurodegeneration and AD pathology in past decades,^2^ accurately predicting rates of cognitive decline in individuals with AD remains challenging.^3^ Given the strong links between tau pathology and key correlates of cognition (eg, neuronal loss and synaptic dysfunction) observed in vitro and at autopsy,^4,5^ in vivo information about the magnitude of cerebral tau pathology might improve the prediction of future cognitive decline.

A variety of positron emission tomography (PET) ligands have been developed that bind with high affinity to the tau aggregates formed in AD.^6,7,8^ The degree and patterns of tau PET retention strongly overlap with regions affected by brain atrophy and hypometabolism^9,10,11,12^ and correlate with concurrent cognitive performance.^13,14,15,16^ In addition, tau PET has shown excellent diagnostic performance for distinguishing AD dementia from non-AD neurodegenerative disorders such as frontotemporal dementia or vascular dementia.^17,18,19,20^ Recently, elevated baseline tau PET levels have been associated with accelerated cognitive decline over time,^21,22,23,24,25,26,27^ but most studies had relatively modest sample sizes, lacked a replication cohort, and/or focused on 1 stage of the AD clinical continuum. The objectives of this prospective, longitudinal multicenter study were to (1) examine the prognostic value of [^18^F]flortaucipir and [^18^F]RO948 tau PET in a large cohort (n = 1431) of individuals with AD dementia, mild cognitive impairment (MCI), or normal cognition; (2) perform a head-to-head comparison of tau PET with established magnetic resonance imaging (MRI) and amyloid PET markers for predicting future cognitive change; and (3) investigate whether age, sex, and/or *APOE* genotype modify the association between baseline tau PET and cognitive change over time.

## Methods

### Participants

From an ongoing multicenter study,^18,28,29,30^ we included 1431 participants from the Memory Disorder Clinic of Gangnam Severance Hospital, Seoul, South Korea (n = 161); the Swedish BioFINDER-1 (n = 136) and BioFINDER-2 (n = 296) studies at Lund University, Lund, Sweden; University of California, San Francisco (UCSF [n = 44]); the Alzheimer Disease Neuroimaging Initiative (ADNI [n = 445]) Avid Radiopharmaceuticals studies (A05 [n = 160]) and the placebo arm of the Eli Lilly solanezumab Expedition-3 study [n = 79]); and the Berkeley Aging Cohort Study (BACS [n = 110]). Data were collected from June 1, 2014, to February 28, 2021. Tau PET was performed using [^18^F]flortaucipir-PET in the discovery cohort (1135 [79.3%] of the total sample) and [^18^F]RO948-PET in the replication cohort (296 [20.7%] of the total sample from BioFINDER-2). Following National Institute on Aging–Alzheimer’s Association diagnostic criteria,^31^ we only included patients with AD dementia who were positive for amyloid-β (Aβ) on PET and/or cerebrospinal fluid (CSF) (n = 315)^18,28,29^; 34 individuals with clinically diagnosed AD dementia who were negative for Aβ were excluded. We also included Aβ-positive (n = 271) and Aβ-negative (n = 172) participants with MCI and Aβ-positive (n = 253) and Aβ-negative (n = 420) cognitively unimpaired individuals (CU group). In addition to tau PET, all participants underwent a medical history assessment and neurological examination, MRI, and a neuropsychological test battery including the Mini-Mental State Examination (MMSE). The MMSE is a diagnostic screening tool that measures a variety of cognitive abilities—including orientation to time and place, short-term episodic memory, attention, problem solving, visuospatial abilities, and language and motor skills—and is often used as a cognitive outcome measure in longitudinal studies and clinical trials. Inclusion criteria for this study were MMSE assessment (n = 1431), MRI scan (n = 1431), and amyloid-PET scan (n = 1329) less than 6 months from tau PET and at least 2 MMSE time points (including baseline) with a minimum follow-up duration of 12 months. Written informed consent was obtained from all participants, and local institutional review boards for human research approved the study. This study followed the Transparent Reporting of a Multivariable Prediction Model for Individual Prognosis or Diagnosis (TRIPOD) reporting guideline.

### PET/MRI Acquisition

We acquired PET images using the following PET/computed tomography (CT) scanners: Biograph mCT (Siemens) in Seoul,^32^ Discovery 690 (GE Healthcare) in BioFINDER-1, Discovery MI (GE Healthcare) in BioFINDER-2,^13,17^ Biograph 6 Truepoint (Siemens) at UCSF and BACS,^12,33^ and multiple scanners in the multicenter ADNI^34^ and Avid Radiopharmaceuticals^23^ cohorts. All PET data were reconstructed at the respective sites into 4 × 5-minute frames within the 80- to 100-minute ([^18^F]flortaucipir) and 70- to 90-minute ([^18^F]RO948) intervals after injection. Amyloid PET was performed using carbon 11 (^11^C)–Pittsburgh Compound B (BACS and UCSF), [^18^F]florbetapir (Avid Radiopharmaceuticals and ADNI subsets), [^18^F]florbetaben (Seoul and ADNI subsets), or [^18^F]flutemetamol (BioFINDER-1 and BioFINDER-2). Magnetic resonance images were acquired on the following scanners: 3.0-T Discovery MR750 (GE Healthcare) in Seoul,^32^ 3.0-T Tim Trio (Siemens) or 3.0-T Prisma (Siemens) in BioFINDER-1 and -2,^13,17^ 3.0-T Tim Trio or 3.0-T Prisma (Siemens) at UCSF,^33^ 1.5-T Magnetom Avanto (Siemens) for BACS,^12^ and multiple 1.5-T and 3-T scanners in the multicenter ADNI^34^ and Avid Radiopharmaceuticals^23^ cohorts.

### T1-Weighted MRI Processing

The MRI data were centrally processed at Lund University using previously reported procedures.^13,17,18,28,29^ Briefly, cortical reconstruction and volumetric segmentation were performed with FreeSurfer, version 6.0, image analysis pipelines (https://surfer.nmr.mgh.harvard.edu/). Magnetization-prepared rapid gradient-echo images underwent correction for intensity homogeneity, removal of nonbrain tissue, and segmentation into gray matter, white matter, and CSF with intensity gradient and connectivity among voxels.^35^ Cortical thickness was measured as the distance from the gray matter–white matter boundary to the perpendicular pial surface.^36^ Reconstructed data sets were visually inspected for accuracy, and segmentation errors were corrected.

### PET Processing

Tau PET images were first resampled to obtain uniform image size (128 × 128 × 63 matrix) and voxel dimensions (2.0 × 2.0 × 2.0 mm) across centers. Next, [^18^F]flortaucipir/[^18^F]RO948 images were centrally processed at Lund University using previously reported procedures,^18,28,29^ followed by motion correction using AFNI’s 3-dimensional volume registration, calculation of mean time, and rigid coregistration to the skull-stripped MRI scan. Voxelwise standardized uptake value ratio (SUVR) images were created using inferior cerebellar gray matter as the reference region.^37^ To extract mean regional SUVR values, FreeSurfer parcellation of the T1-weighted MRI scan was applied to the PET data transformed to participants’ native T1 space. For amyloid PET, we applied computational analysis of PET by AIBL (CapAIBL)^38^ and tracer-specific conversion formulas to convert PET images or SUVR values into a Centiloid scale, which is a standard framework for the quantification of amyloid PET scans across tracers and cohorts.^39^

### Regions of Interest

In line with previous work,^17,18,28^ we calculated the mean [^18^F]flortaucipir and [^18^F]RO948-PET SUVR in the entorhinal cortex,^15,16^ a temporal meta–region of interest (ROI) that is a weighted mean of entorhinal, amygdala, parahippocampal, fusiform, and inferior and middle temporal ROIs,^40^ and Braak stages V to VI encompassing widespread neocortical ROIs.^41^ For MRI, we computed hippocampal volumes (adjusted for intracranial volume), an AD-signature cortical thickness ROI consisting of bilateral entorhinal, inferior, and middle temporal and fusiform cortex^40^ and whole-brain cortical thickness (adjusted for surface area).^40^ The temporal meta-ROI for tau PET and AD-signature cortical thickness ROI for MRI are reported in the main text, whereas the other ROIs are presented in eFigures 2 and 4 in the Supplement.

### Statistical Analyses

We first performed a head-to-head comparison between [^18^F]flortaucipir-PET and MRI for predicting change in MMSE over time. Therefore, single-participant slopes (representing annual change) for MMSE were calculated using linear regression models adjusted for age, sex, educational attainment, and cohort. These slopes were used as dependent variables in linear regression models, including continuous tau PET, MRI, or amyloid PET measures as predictors across the whole group and in the separate diagnostic groups. We performed bootstrapping with 1000 iterations to test whether the *R*^2^ value differed between PET and MRI models. To test whether tau PET and MRI provide complementary information, we applied linear mixed-effects models with random intercepts and fixed slopes using longitudinal MMSE as a dependent variable. Our longitudinal data set was characterized by many participants for whom only 2 MMSE measurements were available. Although linear mixed models are generally able to accommodate this, including random slopes for participants led to overfitting of our models, whereas fixed-participant slopes led to the most parsimonious model. Model 1 included age, sex, educational attainment, and cohort as predictors. In model 2, either baseline tau PET or baseline MRI was added to model 1 as a predictor. In model 3, both imaging modalities (and the predictors from model 1) were entered simultaneously in a single model. We assessed model fit (Akaike information criterion) and examined differences in Akaike information criterion between models 1 and 2 and models 2 and 3 using the χ^2^ statistic. We also performed mediation analysis to examine whether associations between baseline tau PET and longitudinal change in MMSE are mediated by MRI, adjusting for age, sex, educational attainment, cohort, and *APOE*ε4 status. All analyses described above were also performed in the [^18^F]RO948-PET replication cohort and were repeated for a head-to-head comparison between tau PET and amyloid PET (except for the mediation analysis). Finally, we tested whether the association between baseline tau PET and change in MMSE over time across all Aβ-positive participants is moderated by age, sex, or *APOE* genotype using linear mixed-effect models with a 3-way interaction term (time × tau PET × age/sex/*APOE*), adjusted for age, sex, educational attainment, and cohort. Significance level was set at 2-sided *P* < .05. We used R, version 4.0.2 (R Program for Statistical Computing), for the statistical analyses.

## Results

### Participants

Participant characteristics across diagnostic groups are presented in Table 1 (and stratified by discovery/replication sample and by cohort in eTables 1 and 2 in the Supplement, respectively). The mean (SD) age of the study participants was 71.2 (8.8) years; 680 (47.5%) were female and 751 (52.5%) were male. As expected, the AD dementia group had worse baseline MMSE (21.2 [4.2]), annual decline in MMSE (−2.42 [1.87]), and baseline imaging markers (eg, [^18^F]flortaucipir SUVR in the temporal meta-ROI, 1.83 [0.44]), followed by the MCI (baseline MMSE score, 27.0 [2.4]; annual decline in MMSE score, −1.38 [1.84]; [^18^F]flortaucipir SUVR in the temporal meta-ROI, 1.46 [0.36] in Aβ-positive MCI group) and then the CU groups (baseline MMSE score, 28.8 [1.3]; annual decline in MMSE score, −0.37 [0.84]; [^18^F]flortaucipir SUVR in the temporal meta-ROI, 1.22 [0.14] in Aβ-positive CU group). The mean (SD) follow-up duration for MMSE was 1.9 (0.8) years.

**Table 1.** Participant Characteristics^a^

Characteristic	Study group					
	All (N = 1431)	Aβ-positive AD dementia (n = 315)	Aβ-positive MCI (n = 271)	Aβ-negative MCI (n = 172)	Aβ-positive CU (n = 253)	Aβ CU (n = 420)
Age, y	71.2 (8.8)	72.3 (8.4)	71.7 (7.9)	70.1 (8.2)	73.6 (7.2)	69.1 (10.0)
Sex, %						
Male	52.5	58.4	50.6	45.9	49.4	53.8
Female	47.5	41.6	49.4	54.1	50.6	46.2
Educational attainment, y	13.4 (6.0)	12.5 (5.0)	12.2 (5.2)	12.5 (5.5)	15.5 (8.8)	14.0 (5.0)
*APOE* ε4-positive, No./total No. (%)	616/1378 (44.7)	200/296 (67.6)	162/261 (62.1)	34/166 (20.5)	137/247 (55.5)	83/408 (20.3)
MMSE, baseline score	26.7 (3.9)	21.2 (4.2)	27.0 (2.4)	28.0 (1.9)	28.8 (1.3)	29.0 (1.2)
MMSE, annual change	–1.01 (1.61)	–2.42 (1.87)	–1.38 (1.84)	–0.74 (1.31)	–0.37 (0.84)	–0.19 (0.55)
Follow-up duration, mo	22.7 (9.8)	19.8 (10.2)	22.8 (10.4)	20.8 (9.0)	24.0 (10.1)	24.6 (8.8)
Follow-up visits, median (range)	2 (2-6)	2 (2-5)	3 (2-5)	2 (2-5)	3 (2-5)	2 (2-5)
[^18^F]flortaucipir/[^18^F]RO948, No. of participants	1135/296	235/80	190/81	144/28	208/45	358/62
Flortaucipir temporal meta-ROI, SUVR	1.39 (0.38)	1.83 (0.44)	1.46 (0.36)	1.18 (0.12)	1.22 (0.14)	1.17 (0.09)
RO948 temporal meta-ROI, SUVR	1.49 (0.57)	2.15 (0.65)	1.35 (0.32)	1.16 (0.10)	1.24 (0.25)	1.14 (0.07)
AD-signature cortical thickness, mm	2.63 (0.22)	2.40 (0.20)	2.60 (0.20)	2.68 (0.20)	2.72 (0.17)	2.76 (0.15)
Amyloid PET/CSF Aβ findings, No. of participants	1329/102	224/91	264/7	170/2	252/1	419/1
Amyloid PET, Centiloids	43.4 (47.7)	95.5 (33.9)	77.0 (36.2)	–0.6 (11.6)	57.7 (34.8)	2.4 (9.8)

Abbreviations: Aβ, amyloid-β; AD, Alzheimer disease; *APOE,* apolipoprotein E; CSF, cerebrospinal fluid; CU, cognitively unimpaired; MCI, mild cognitive impairment; MMSE, Mini-Mental State Examination; PET, positron emission tomography; ROI, region of interest; SUVR, standardized uptake value ratio.

^a^ Unless otherwise indicated, data are expressed as mean (SD).

### Head-to-Head Comparison: Tau PET vs MRI

**Figure 1.**
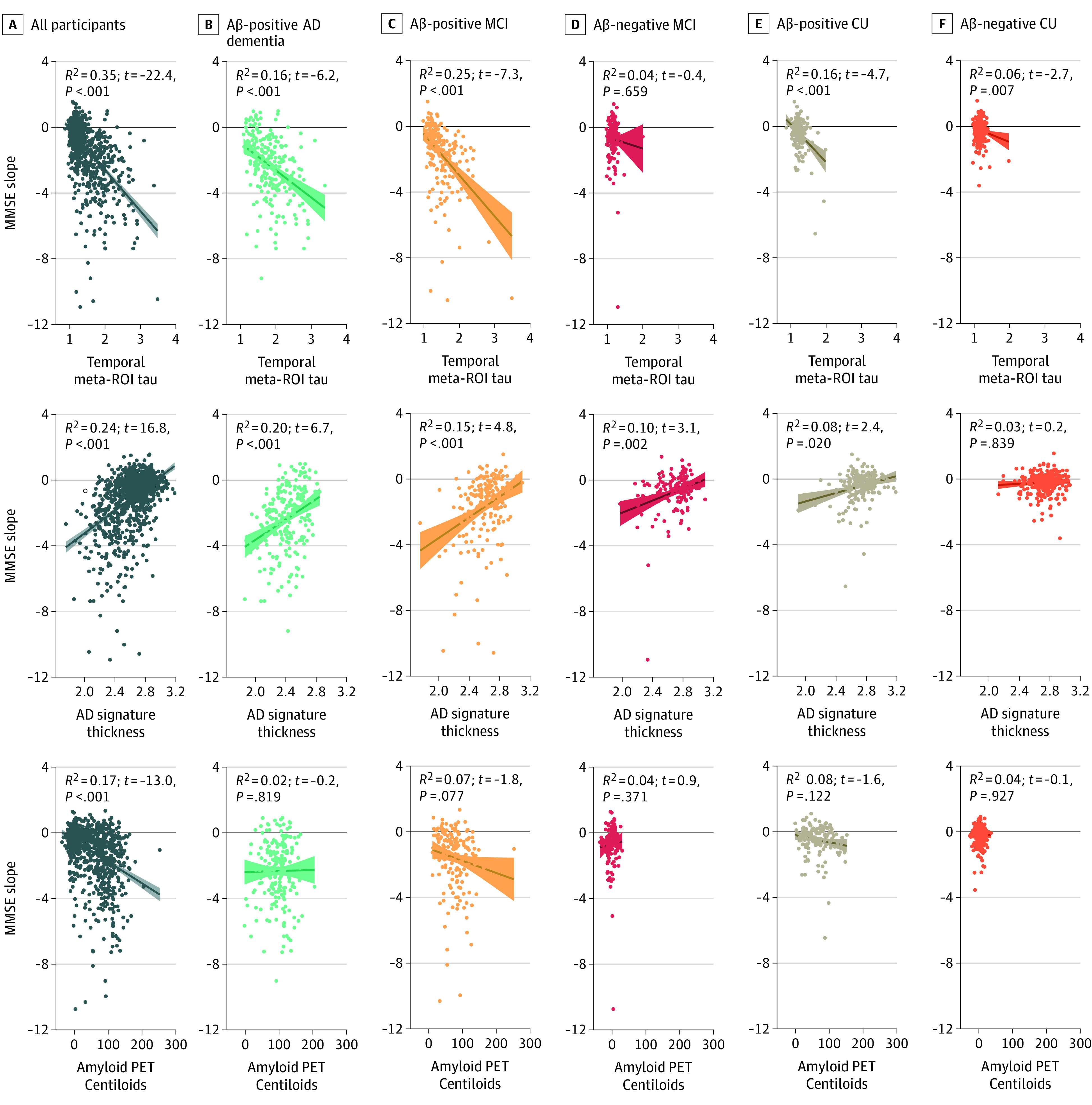
Association of Baseline Tau Positron Emission Tomography (PET), Magnetic Resonance Imaging (MRI), and Amyloid PET With Change in Mini-Mental State Examination (MMSE) Graphs represent associations between baseline fluorine 18–labeled flortaucipir (tau) PET uptake in a temporal region of interest (top row), cortical thickness in an Alzheimer disease (AD) signature region defined on MRI (middle row), and amyloid PET (bottom row) with annual slopes of MMSE scores across all participants (A), the amyloid-β (Aβ)-positive AD dementia group (B), the Aβ-positive mild cognitive impairment (MCI) group (C), the Aβ-negative MCI group (D), the Aβ-positive cognitively unimpaired (CU) group (E), and the Aβ-negative CU group (F). Model outputs are derived from a linear regression model between baseline tau PET/MRI/amyloid PET and MMSE slopes, adjusted for age, sex, educational attainment, and cohort. *R*^2^ values are provided for the full model (including covariates), and *t* test and *P* values represent the interaction between the imaging modality and time.

When comparing [^18^F]flortaucipir SUVR in the temporal meta-ROI against MRI-based AD-signature cortical thickness in linear regression models with annual change in MMSE as dependent variable (Figure 1 and eTable 3 in the Supplement), greater [^18^F]flortaucipir uptake was more strongly associated with decline in MMSE over time than MRI across all participants (*R*^2^, 0.35 [tau PET] vs 0.24 [MRI]; bootstrapped *R*^2^ difference, *t* = 80.3 [*P* < .001]), the Aβ-positive MCI group (R^2^, 0.25 [tau PET] vs 0.15 [MRI]; bootstrapped *R*^2^ difference, *t* = 30.8 [*P* < .001]), the Aβ-positive CU group (*R*^2^, 0.16 [tau PET] vs 0.08 [MRI]; bootstrapped *R*^2^ difference, *t* = 38.6 [*P* < .001]), and the Aβ-negative CU group (*R*^2^, 0.06 [tau PET] vs 0.03 [MRI]; bootstrapped *R*^2^ difference, *t* = 13.6 [*P* < .001]). Magnetic resonance imaging performed better than tau PET in the Aβ-negative MCI (*R*^2^, 0.04 [tau PET] vs 0.10 [MRI]; bootstrapped *R*^2^ difference, *t* = −114.0 [*P* < .001]) and AD dementia (*R*^2^, 0.16 [tau PET] vs 0.20 [MRI]; bootstrapped *R*^2^ difference, *t* = −17.2 [*P* < .001]) groups. Comparable results were found in the [^18^F]RO948 replication cohort (eFigure 1 and eTable 3 in the Supplement), with greater tau PET uptake being more strongly associated with annual decline in MMSE than MRI across all participants (*R*^2^, 0.49 vs 0.34; bootstrapped *R*^2^ difference, *t* = 147.9 [*P* < .001]), the Aβ-positive MCI group (*R*^2^, 0.34 vs 0.20; bootstrapped *R*^2^ difference, *t* = 23.1 [*P* < .001]), the Aβ-positive CU group (*R*^2^, 0.53 vs 0.36; bootstrapped *R*^2^ difference, *t* = 16.7 [*P* < .001]), and the Aβ-negative CU group (*R*^2^, 0.04 vs 0.03; bootstrapped *R*^2^ difference, *t* = 15.4 [*P* < .001]) and with better performance for MRI compared with PET in the Aβ-negative MCI group (*R*^2^, 0.15 vs 0.16; bootstrapped *R*^2^ difference, *t* = −61.4 [*P* < .001]). Contrary to the discovery cohort, in the AD dementia group, [^18^F]RO948 SUVR was more strongly associated with MMSE change (*R*^2^, 0.26 vs 0.17; bootstrapped *R*^2^ difference, *t* = 50.6 [*P* < .001]). In sensitivity analyses assessing entorhinal and Braak stages V and VI ROIs, tau PET was more strongly associated with MMSE change than MRI across all participants, the Aβ-positive MCI group, and the Aβ-positive CU group (eFigures 2-5 in the Supplement).

### Complementary Information by PET and MRI

The results presented in Table 2 indicate that the prediction of decline in MMSE over time improved with both tau PET (*R*^2^ for all participants, 0.49; *R*^2^ for Aβ-positive AD dementia group, 0.34; *R*^2^ for Aβ-positive MCI group, 0.35; *R*^2^ for Aβ-positive CU group, 0.17) and MRI (*R*^2^ for all participants, 0.46; *R*^2^ for Aβ-positive AD dementia group, 0.38; *R*^2^ for Aβ-positive MCI group, 0.29; *R*^2^ for Aβ-positive CU group, 0.12) compared with a basic model including age, sex, educational attainment, and cohort (*R*^2^ for all participants, 0.19; *R*^2^ for Aβ-positive AD dementia group, 0.20; *R*^2^ for Aβ-positive MCI group, 0.21; *R*^2^ for Aβ-positive CU group, 0.08) (all *P* < .001). Furthermore, tau PET and MRI provide complementary information, because when adding tau PET to linear mixed-effects models assessing MRI measures, the *R*^2^ value increased (all participants, 0.46 vs 0.55; Aβ-positive AD dementia group, 0.38 vs 0.41; Aβ-positive MCI group, 0.29 vs 0.36; Aβ-positive CU group, 0.12 vs 0.18) and Akaike information criterion decreased (all participants, 8309 vs 8166; Aβ-positive AD dementia group, 3266 vs 3251; Aβ-positive MCI group, 2904 vs 2873; Aβ-positive CU group, 1922 vs 1901), and vice versa (*R*^2^: 0.49 vs 0.56 for all participants, 0.34 vs 0.43 for Aβ-positive AD dementia group, 0.35 vs 0.39 for Aβ-positive MCI group, and 0.17 vs 0.19 for Aβ-positive CU group; Akaike information criterion: 8188 vs 8085 for all participants, 3265 vs 3224 for Aβ-positive AD dementia group, 2852 vs 2838 for Aβ-positive MCI group, and 1902 vs 1896 for Aβ-positive CU group) (all *P* < .001). Adding [^18^F]flortaucipir-PET to MRI models improved model fit to a larger extent than adding AD-signature cortical thickness to PET models in the total group (χ^2^, 146.0 vs 115.0), Aβ-positive MCI group (χ^2^, 37.2 vs 19.7), and Aβ-positive CU group (χ^2^, 22.6 vs 7.9), but not the AD dementia group (χ^2^, 17.1 vs 42.7). In the replication cohort (eTable 4 in the Supplement), [^18^F]RO948 temporal meta-ROI SUVR always provided complementary information to models that only included AD-signature cortical thickness (all participants, χ^2^ = 176.2; AD dementia group, χ^2^ = 22.9; Aβ-positive MCI group, χ^2^ = 11.2; and Aβ-positive CU group, χ^2^ = 21.3 [*P* < .001]). Magnetic resonance imaging provided complementary information to PET models when including all participants (χ^2^ = 107.8), the AD dementia group (χ^2^ = 6.6), and the Aβ-positive MCI group (χ^2^ = 1.6 [all *P* < .001]), but not the Aβ-positive CU group (χ^2^ = 1.0 [*P* = .31]).

**Table 2.** Complementary Information Provided by Tau PET and MRI for Predicting Change in MMSE^a^

Model by study group	β (SE)	*P* value	*R*^2^ value	AIC	χ^2^ For difference	*P* value for difference
**All Aβ-positive participants**						
Model 1: age, sex, educational attainment, cohort			0.192	8678		
Model 2: model 1 plus tau PET	–0.21 (0.02)	<.001	0.494	8188	483.9	<.001
Model 3: model 1 plus tau PET plus MRI	–0.21 (0.02)	<.001	0.561	8085	115.0	<.001
Model 2: model 1 plus MRI	0.27 (0.03)	<.001	0.463	8309	372.3	<.001
Model 3: model 1 plus MRI plus tau PET	0.27 (0.03)	<.001	0.546	8166	146.0	<.001
**Aβ-positive AD dementia group**						
Model 1: age, sex, educational attainment, cohort			0.202	3349		
Model 2: model 1 plus tau PET	–0.17 (0.03)	<.001	0.337	3265	88.4	<.001
Model 3: model 1 plus tau PET plus MRI	–0.17 (0.03)	<.001	0.425	3224	42.7	<.001
Model 2: model 1 plus MRI	0.22 (0.06)	<.001	0.384	3266	87.2	<.001
Model 3: model 1 plus MRI plus tau PET	0.23 (0.06)	<.001	0.414	3251	17.1	<.001
**Aβ-positive MCI group**						
Model 1: age, sex, educational attainment, cohort	NA	NA	0.212	2945	NA	NA
Model 2: model 1 plus tau PET	–0.25 (0.03)	<.001	0.346	2852	92.9	<.001
Model 3: model 1 plus tau PET plus MRI	–0.26 (0.03)	<.001	0.390	2838	19.7	<.001
Model 2: model 1 plus MRI	0.23 (0.05)	<.001	0.288	2904	41.0	<.001
Model 3: model 1 plus MRI plus tau PET	0.24 (0.05)	<.001	0.356	2873	37.2	<.001
**Aβ-positive CU group**						
Model 1: age, sex, educational attainment, cohort	NA	NA	0.076	1933	NA	NA
Model 2: model 1 plus tau PET	–0.18 (0.05)	<.001	0.167	1902	35.3	<.001
Model 3: model 1 plus tau PET plus MRI	–0.18 (0.05)	<.001	0.188	1896	7.9	.005
Model 2: model 1 plus MRI	0.10 (0.04)	.005	0.117	1922	15.8	<.001
Model 3: model 1 plus MRI plus tau PET	0.10 (0.04)	.005	0.180	1901	22.6	<.001

Abbreviations: Aβ, amyloid-β; AD, Alzheimer disease; AIC, Akaike information criterion; CU, cognitively unimpaired; MCI, mild cognitive impairment; MMSE, Mini-Mental State Examination; MRI, magnetic resonance imaging; NA, not available; PET, positron emission tomography.

^a^ In this analysis, we used the temporal meta–region of interest (ROI) for [^18^F]flortaucipir (tau) PET and AD-signature cortical thickness as an MRI marker as predictors of change in MMSE scores. Reported values represent outputs from linear mixed-effects models with random intercepts and fixed slopes (β [SE] and *R*^2^ value) and from analysis of variance comparing different models (AIC and χ^2^). The β (SE) values represent the interaction between the imaging modality and time; (marginal) *R*^2^ value represents the explained variance by the fixed effects; and AIC represents the model fit. The χ^2^ for difference compares a model with a less advanced model (thus model 2 vs model 1, and model 3 vs model 2).

### Mediation Analyses

**Figure 2.**
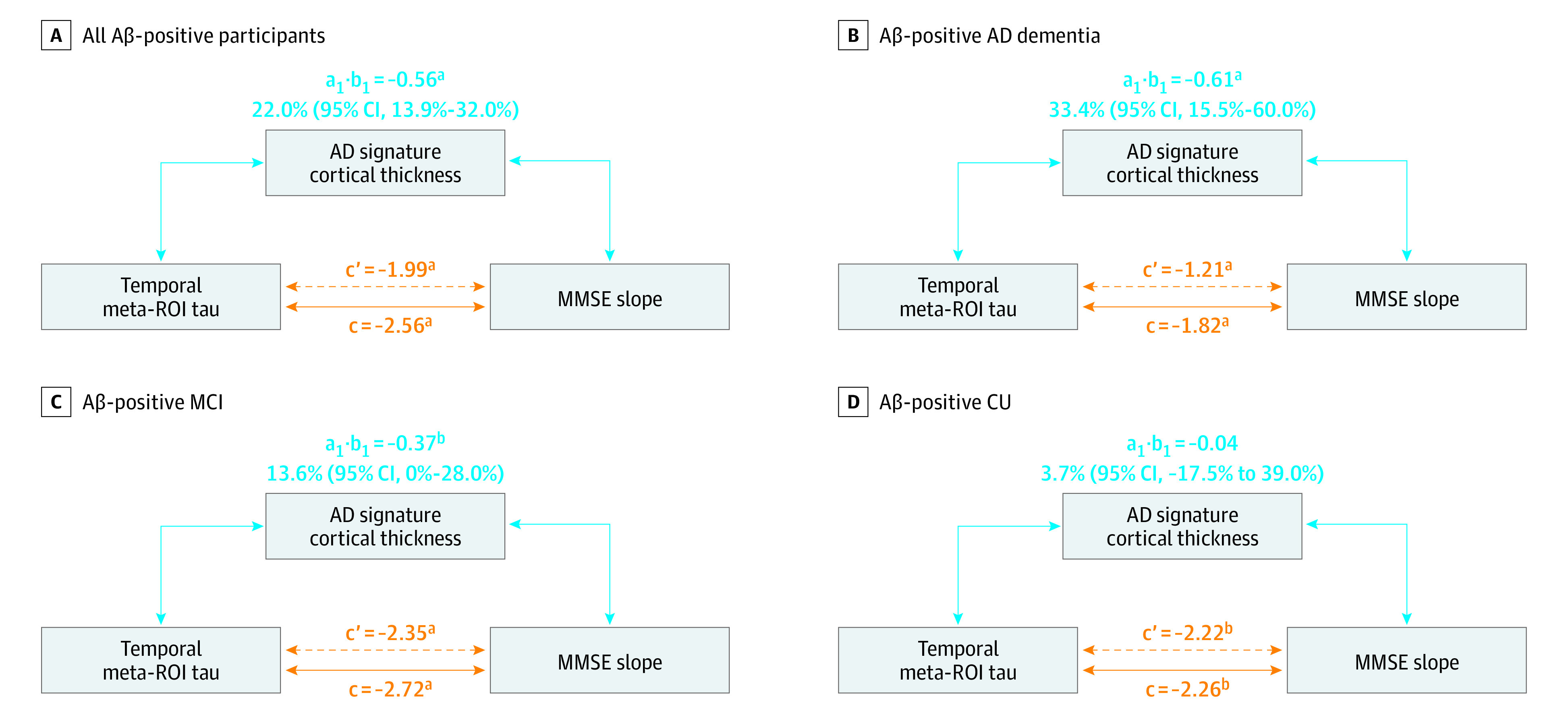
Mediation Analyses Path diagrams indicate whether Alzheimer disease (AD)–signature cortical thickness mediates the associations between baseline fluorine 18–labeled flortaucipir standardized uptake value ratio (SUVR) in the temporal meta–region of interest (ROI) and Mini-Mental State Examination (MMSE) slopes, adjusted for age, sex, educational level, cohort, and *APOE* ε4 status. The direct effect (ie, coefficient c') reflects the extent to which MMSE slopes change when baseline tau positron emission tomography (PET) increases by 1 unit while baseline cortical thickness remains unaltered. The indirect effect (ie, coefficient a_1_ × b_1_) reflects the extent to which MMSE slopes change when baseline tau PET is held constant and baseline cortical thickness changes by the amount it would have changed had baseline tau PET increased by 1 unit. The coefficient c represents the total effect (ie, direct plus indirect effects). Aβ indicates amyloid-β; CU, cognitively unimpaired; MCI, mild cognitive impairment. ^a^*P* < .001. ^b^*P* < .05.

Figure 2 shows path diagrams assessing AD-signature cortical thickness as a potential mediator of associations between baseline [^18^F]flortaucipir temporal meta-ROI SUVR and MMSE slopes. There was a modest mediation effect in the total group (22.0% [95% CI, 13.9%-32.0%] of the total effect; *P* < .001), the AD dementia group (33.4% [95% CI, 15.5%-60.0%] of the total effect; *P* < .001), and the Aβ-positive MCI group (13.6% [95% CI, 0.0%-28.0%] of the total effect; *P* = .04), but not in the Aβ-positive CU group (3.7% [95% CI, −17.5% to 39.0%]; *P* = .71). In the replication cohort (eFigure 6 in the Supplement), the association between baseline [^18^F]RO948 SUVR and decline in MMSE was only modestly mediated by AD-signature cortical thickness across all participants (21.0% [95% CI, 9.8%-35.0%]; *P* < .001), but not in the AD dementia (13.0% [95% CI, −0.5% to 41.0%]; *P* = .06), Aβ-positive MCI (9.0% [95% CI, −8.0% to 54.0%]; *P* = .24), and Aβ-positive CU (19.8% [95% CI, −50.9% to 56.0%]; *P* = .33) groups.

### Head-to-Head Comparison: Tau PET vs Amyloid PET

Figure 1 and eTable 5 in the Supplement indicate that [^18^F]flortaucipir-PET was more strongly associated with annual decline in MMSE than amyloid PET across all participants (*R*^2^, 0.35 vs 0.17; bootstrapped *R*^2^ difference, *t* = 147.1 [*P* < .001]), the AD dementia group (*R*^2^, 0.17 vs 0.02; bootstrapped *R*^2^ difference, *t* = 81.1 [*P* < .001]), the Aβ-positive MCI group (*R*^2^, 0.25 vs 0.07; bootstrapped *R*^2^ difference, *t* = 63.3 [*P* < .001]), the Aβ-positive CU group (*R*^2^, 0.16 vs 0.08; bootstrapped *R*^2^ difference, *t* = 47.0 [*P* < .001]), and the Aβ-negative CU group (*R*^2^, 0.06 vs 0.04; bootstrapped *R*^2^ difference, *t* = 21.7 [*P* < .001]). Comparable results were found in the [^18^F]RO948 replication cohort (eFigure 1 and eTable 5 in the Supplement). Tau PET always added information to models including amyloid PET (*R*^2^ for all participants, 0.49 vs 0.25; *R*^2^ for Aβ-positive AD dementia group, 0.33 vs 0.20; *R*^2^ for Aβ-positive MCI group, 0.39 vs 0.24; *R*^2^ for Aβ-positive CU group, 0.18 vs 0.11) (all *P* < .001) (eTable 6 in the Supplement), whereas amyloid PET did not improve tau PET models in the AD dementia (χ^2^, 0.1 [*P* = .82]), Aβ-positive MCI (χ^2^, 0.01 [*P* = .97]), and Aβ-positive CU (χ^2^, 0.2 [*P* = .69]) groups.

### Modification of Tau PET vs Cognitive Decline Associations by Age, Sex, and *APOE* Genotype

**Figure 3.**
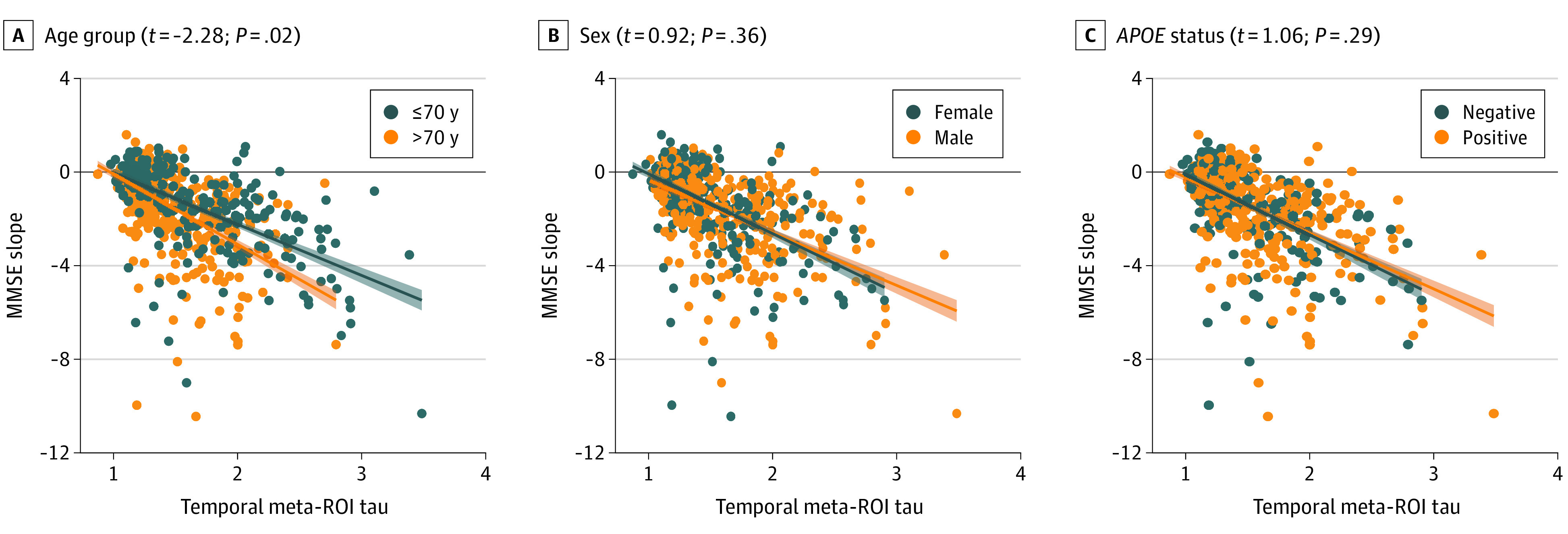
Age, Sex, and *APOE* ε4 Status as Potential Modifiers of the Association Between Baseline Tau Positron Emission Tomography (PET) and Cognitive Change Over Time Linear mixed-effects models with random intercepts and fixed slopes were performed to examine whether age, sex, and *APOE* ε4 status moderate the association between baseline fluorine 18–labeled flortaucipir uptake in a temporal region of interest (ROI) and change over time in Mini-Mental State Examination (MMSE) scores while adjusting for age, sex, educational attainment, cohort, and diagnostic group when appropriate. The *t* tests and *P* values represent the 3-way interaction of age/sex/*APOE* ε4 status × time × tau PET. Age was entered as continuous variable in the linear mixed-effects models but was dichotomized at 70 years for visualization purposes.

Linear mixed-effects models showed that age (*t* = −2.28; *P* = .02), but not sex (*t* = 0.92; *P* = .36) or *APOE* genotype (*t* = 1.06; *P* = .29), modified the association between baseline [^18^F]flortaucipir temporal meta-ROI SUVR and MMSE change, because older individuals showed faster cognitive decline at similar tau PET levels (Figure 3). In the [^18^F]RO948 cohort, modification by age was not replicated (*t* = −0.81; *P* = .42) (eFigure 7 in the Supplement). Consistent with the discovery cohort, there were no significant 3-way interactions for sex (*t* = −1.67; *P* = .10) and *APOE* genotype (*t* = −0.47; *P* = .64).

## Discussion

The main finding of this multicenter prognostic study was that baseline tau PET predicts group-level changes in MMSE over time across the AD clinical spectrum. In a head-to-head comparison with established MRI and amyloid PET markers, tau PET showed stronger associations with cognitive change, especially in preclinical and prodromal stages of AD. Part of the association between baseline tau PET and cognitive decline over time was mediated by baseline cortical thickness, but tau PET and MRI also provided complementary prognostic information. We identified age as a potential moderator of the association between baseline tau PET and longitudinal cognitive change, because older individuals showed more rapid cognitive decline at similar levels of tau load compared with younger individuals. Altogether, our findings suggest that tau PET is a promising tool for predicting future cognitive change that could support the prognostic process, especially in preclinical and prodromal stages of AD.

Clinicopathological studies^4,5^ have identified strong associations between tau pathology and cognition as well as key correlates of cognition such as loss of neurons and synaptic activity. These observations have been replicated in vivo using PET ligands that detect neocortical AD-like tau pathology with high accuracy,^7^ because increased tau PET retention was associated with worse concurrent cognitive performance^10,14,15,16^ as well as reductions in gray matter volume, glucose metabolism, and synaptic density.^9,42,43^ Recent studies have indicated that elevated baseline tau PET levels were associated with accelerated cognitive decline over time,^21,22,23,24,25,26,27^ but most of these studies had relatively modest sample sizes, included retrospective cognitive time points, lacked a replication cohort focused on only 1 stage of the AD clinical continuum, and/or did not perform head-to-head comparisons against MRI and amyloid PET markers. We included a large study population with prospective longitudinal assessment of MMSE across the clinical AD spectrum and demonstrate that tau PET is a powerful predictor of cognitive change over time and outperformed MRI and amyloid PET markers. This is an important first step toward further investigation of the potential of tau PET to act as a prognostic marker, especially in the early stages of AD, when estimating rates of future decline is notoriously challenging. Future research directions include the use of more sensitive (eg, the preclinical Alzheimer cognitive composite) or domain-specific (eg, episodic memory or executive functioning) cognitive tests, functional measures (eg, Clinical Dementia Rating Scale Sum of Boxes) or diagnostic conversion (eg, from MCI to AD dementia) as clinical readouts, longer follow-up durations, assessment of individualized prognostic models, and head-to-head comparisons against fluid biomarkers (eg, plasma phosphorylated tau) that are more scalable and possibly more cost-effective. Furthermore, in a recent successful phase 2 clinical trial with the Aβ-antibody donanemab,^44^ Aβ-positive individuals with MCI or mild dementia were specifically selected based on intermediate levels of tau pathology on a PET scan. This suggests that tau PET biomarkers could be used as a selection tool for trial participants, but further investigation is warranted.

We found that tau PET outperformed MRI markers in predicting future cognitive decline across all participants, in the Aβ-positive MCI group, and in the Aβ-positive CU group (both in the discovery and replication cohorts). For AD dementia, the results were inconsistent, with MRI performing slightly better compared with [^18^F]flortaucipir in the discovery cohort, whereas in the replication sample, [^18^F]RO948-PET clearly outperformed MRI. Because there were no major demographic differences between AD cases in the discovery vs the replication cohort (eTable 1 in the Supplement), this discrepant finding may be explained by the slightly greater dynamic range of [^18^F]RO948 compared with [^18^F]flortaucipir that enables [^18^F]RO948 to slightly better capture cognitive change over time in more advanced clinical stages of AD. Altogether, these findings are in line with those of a previous cross-sectional study^13^ showing that tau PET is more sensitive than MRI for detecting early cognitive change, whereas at the dementia stage, tau PET and MRI perform more equally. Greater sensitivity to detect early cognitive change using tau PET compared with MRI can possibly be explained by the large variations in brain structure that preexist in the general population, which may reduce the ability of structural MRI to reliably distinguish the earliest AD-related changes from premorbid differences in brain structure accentuated by age-related brain changes. Furthermore, tau PET may be more sensitive to early changes owing to the presumed occurrence of tau pathology before onset of neurodegeneration,^45^ which might affect cognition through both structural (brain atrophy)^46^ and functional (network disruption)^47^ pathways. Finally, we found that cortical thickness only modestly mediated the association between baseline tau PET and MMSE slopes, an effect that was disease-stage specific because it was only observed in the AD dementia and Aβ-positive MCI groups (discovery cohort only, not replicated), but not in the Aβ-positive CU group. Tau PET also outperformed amyloid PET in predicting future cognitive change, which is in accordance with previous observations of modest cognitive correlates for levels of Aβ in stark contrast to associations of pathological tau burden.^4,10,14,15,16,23,24,25^ This can be explained by differences in the temporal evolution of Aβ and tau pathology. Widespread Aβ pathology may emerge approximately 20 years before symptom onset, but the rate of accumulation attenuates over the disease course, which reduces its clinicopathological correlates.^4,48^ In contrast, neocortical tau pathology is typically only observed when the disease has clinically manifested, and rates of tau accumulation are higher in symptomatic compared with asymptomatic individuals on the AD pathological continuum.^18,49^ Overall, our findings suggest that tau PET may be the most optimal biomarker to identify those Aβ-positive individuals who are at risk for future cognitive decline and to predict cognitive trajectories in clinical trial participants with preclinical or prodromal AD.

Age, sex, and *APOE* genotype have previously been shown to affect rates of tau accumulation and cognitive performance across the AD clinical spectrum.^50,51,52^ In the present study, we examined whether age, sex, and *APOE* genotype act as modifiers of the association between baseline tau PET and cognitive change over time. In the discovery cohort, older individuals showed more rapid cognitive decline than younger individuals with a similar tau load. This could be explained by lower resilience against tau pathology (and/or associated neurodegeneration) in older individuals or by the presence of co–pathological features (eg, TAR DNA-binding protein 43 or vascular pathology) that are more likely to occur with advancing age. Sex did not affect the association between baseline tau PET and cognitive change over time. Previous work has suggested that this effect may only pertain to preclinical AD, wherein women showed faster rates of cognitive decline at similar (high) levels of tau pathology compared with men.^53^ The association between baseline tau PET and cognitive change over time did not differ by *APOE* genotype.

### Strengths and Limitations

The strengths of this study include the large sample size, coverage of the full AD clinical spectrum, and availability of tau PET, MRI, amyloid PET, and prospective longitudinal MMSE scores. There are also several limitations. First, MMSE served as an outcome measure because it is the only cognitive test available across all cohorts in this study. Although MMSE is a widely used measure in clinical practice and clinical trials, it is a relatively crude measure that is characterized by a ceiling effect, and the follow-up duration of this study was relatively short. Second, inherent to multicenter studies comprising multiple cohorts that were not codesigned at inception, several challenges exist regarding data harmonization and pooling. Moreover, additional complexities exist related to use of different criteria for study entry and differences in clinical assessment at each site. Similar to previous studies using this sample,^18,28,29,30^ we minimized variability by analyzing data centrally at Lund University using a uniform pipeline, and we adjusted for cohort effects in the statistical models. However, dissimilarities in participant selection, data acquisition, and preprocessing remain. Third, despite geographical contributions from Europe, Asia, and the US, most study participants were non-Hispanic White individuals. Future studies should test whether the study findings are generalizable to more ethnically diverse populations. Fourth, we used a different tau PET tracer in the replication cohort, informed by previous studies demonstrating good correspondence between [^18^F]flortaucipir-PET and [^18^F]RO948-PET for neocortical tracer uptake and tau PET positivity rates.^28,54^

## Conclusions

In this multicenter prognostic study, the tau PET tracers [^18^F]flortaucipir and [^18^F]RO948 demonstrated prognostic utility as strong predictors of cognitive change over time. Tau PET outperformed established MRI and amyloid PET markers in a head-to-head comparison, especially in the Aβ-positive MCI and Aβ-positive CU groups. Our findings suggest that although tau PET as a diagnostic marker is most valuable at the dementia stage of AD,^17,18,20^ the optimal time window for tau PET as a prognostic marker is during the prodromal and preclinical stages of AD.

## Notes

Supplement. **eFigure 1.** Replication of Figure 1 (PET vs MRI Comparison) Using [^18^F]RO948 PET in an Independent Dataset **eFigure 2.** [^18^F]Flortaucipir SUVR in Entorhinal Cortex and Hippocampal Volumes as Predictors of Change in MMSE **eFigure 3.** Replication of eFigure 2 With [^18^F]RO948 PET in an Independent Dataset **eFigure 4.** [^18^F]Flortaucipir SUVR in Braak V/VI ROIs and Whole Brain Cortical Thickness as Predictors of Change in MMSE **eFigure 5.** Replication of eFigure 4 With [^18^F]RO948 PET in an Independent Dataset **eFigure 6.** Replication of Figure 2 (Mediation Analysis) Using [^18^F]RO948 PET in an Independent Dataset **eFigure 7.** Replication of Figure 3 (Associations With Age, Sex, and APOE) Using [^18^F]RO948 PET in an Independent Dataset **eTable 1.** Participant Characteristics for the Discovery Cohort (A) vs the Replication Cohort (B) **eTable 2.** Participant Characteristics for the Different Cohorts **eTable 3.** Bootstrapping of *R*^2^ Values From Tau PET vs MRI Models Predicting MMSE Change **eTable 4.** Linear Mixed Models With [^18^F]RO948 PET and MRI as Predictors of Change in MMSE **eTable 5.** Bootstrapping of *R*^2^ Values From Tau PET vs Amyloid PET Models Predicting MMSE Change **eTable 6.** Linear Mixed Models With [^18^F]Flortaucipir PET and Amyloid PET as Predictors of Change in MMSE **Group Information.** ADNI Investigators
