# Supplementary material for: Accuracy of Tau Positron Emission Tomography as a Prognostic Marker in Preclinical and Prodromal Alzheimer Disease: A Head-to-Head Comparison Against Amyloid Positron Emission Tomography and Magnetic Resonance Imaging
Source: JAMA Neurol. 2021 Jun 28;78(8):961–71. doi: 10.1001/jamaneurol.2021.1858 (PMC8240013; doi:10.1001/jamaneurol.2021.1858)
Supplement: Supplement. — eFigure 1. Replication of Figure 1 (PET vs MRI Comparison) Using [18F]RO948 PET in an Independent Dataset eFigure 2. [18F]Flortaucipir SUVR in Entorhinal Cortex and Hippocampal Volumes as Predictors of Change in MMSE eFigure 3. Replication of eFigure 2 With [18F]RO948 PET in an Independent Dataset eFigure 4. [18F]Flortaucipir SUVR in Braak V/VI ROIs and Whole Brain Cortical Thickness as Predictors of Change in MMSE eFigure 5. Replication of eFigure 4 With [18F]RO948 PET in an Independent Dataset eFigure 6. Replication of Figure 2 (Mediation Analysis) Using [18F]RO948 PET in an Independent Dataset eFigure 7. Replication of Figure 3 (Associations With Age, Sex, and APOE) Using [18F]RO948 PET in an Independent Dataset eTable 1. Participant Characteristics for the Discovery Cohort (A) vs the Replication Cohort (B) eTable 2. Participant Characteristics for the Different Cohorts eTable 3. Bootstrapping of R2 Values From Tau PET vs MRI Models Predicting MMSE Change eTable 4. Linear Mixed Models With [18F]RO948 PET and MRI as Predictors of Change in MMSE eTable 5. Bootstrapping of R2 Values From Tau PET vs Amyloid PET Models Predicting MMSE Change eTable 6. Linear Mixed Models With [18F]Flortaucipir PET and Amyloid PET as Predictors of Change in MMSE Group Information. ADNI Investigators [file jamaneurol-e211858-s001.pdf]

# Supplementary Online Content

Ossenkoppele R, Smith R, Mattsson-Carlgren N, et al. Accuracy of tau positron emission tomography as a prognostic marker in preclinical and prodromal Alzheimer disease: a head-to-head comparison against amyloid positron emission tomography and magnetic resonance imaging. *JAMA Neurol*. Published online June 28, 2021. doi:10.1001/jamaneurol.2021.1858


**eFigure 1.** Replication of Figure 1 (PET vs MRI Comparison) Using [$^{18}$F]RO948 PET in an Independent Dataset
**eFigure 2.** [$^{18}$F]Flortaucipir SUVR in Entorhinal Cortex and Hippocampal Volumes as Predictors of Change in MMSE
**eFigure 3.** Replication of eFigure 2 With [$^{18}$F]RO948 PET in an Independent Dataset
**eFigure 4.** [$^{18}$F]Flortaucipir SUVR in Braak V/VI ROIs and Whole Brain Cortical Thickness as Predictors of Change in MMSE
**eFigure 5.** Replication of eFigure 4 With [$^{18}$F]RO948 PET in an Independent Dataset
**eFigure 6.** Replication of Figure 2 (Mediation Analysis) Using [$^{18}$F]RO948 PET in an Independent Dataset
**eTable 1.** Participant Characteristics for the Discovery Cohort (A) vs the Replication Cohort (B)
**eTable 2.** Participant Characteristics for the Different Cohorts
**eTable 3.** Bootstrapping of $R^2$ Values From Tau PET vs MRI Models Predicting MMSE Change
**eTable 4.** Linear Mixed Models With [$^{18}$F]RO948 PET and MRI as Predictors of Change in MMSE
**eTable 5.** Bootstrapping of $R^2$ Values From Tau PET vs Amyloid PET Models Predicting MMSE Change
**eTable 6.** Linear Mixed Models With [$^{18}$F]Flortaucipir PET and Amyloid PET as Predictors of Change in MMSE
**eFigure 7.** Replication of Figure 3 (Associations With Age, Sex, and APOE) Using [$^{18}$F]RO948 PET in an Independent Dataset
**eAppendix.** ADNI Investigators


This supplementary material has been provided by the authors to give readers additional information about their work.

© 2021 Ossenkoppele R et al. *JAMA Neurology*.

eFigure 1. Replication of Figure 1 (PET vs MRI Comparison) Using [$^{18}$F]RO948 PET in an Independent Dataset

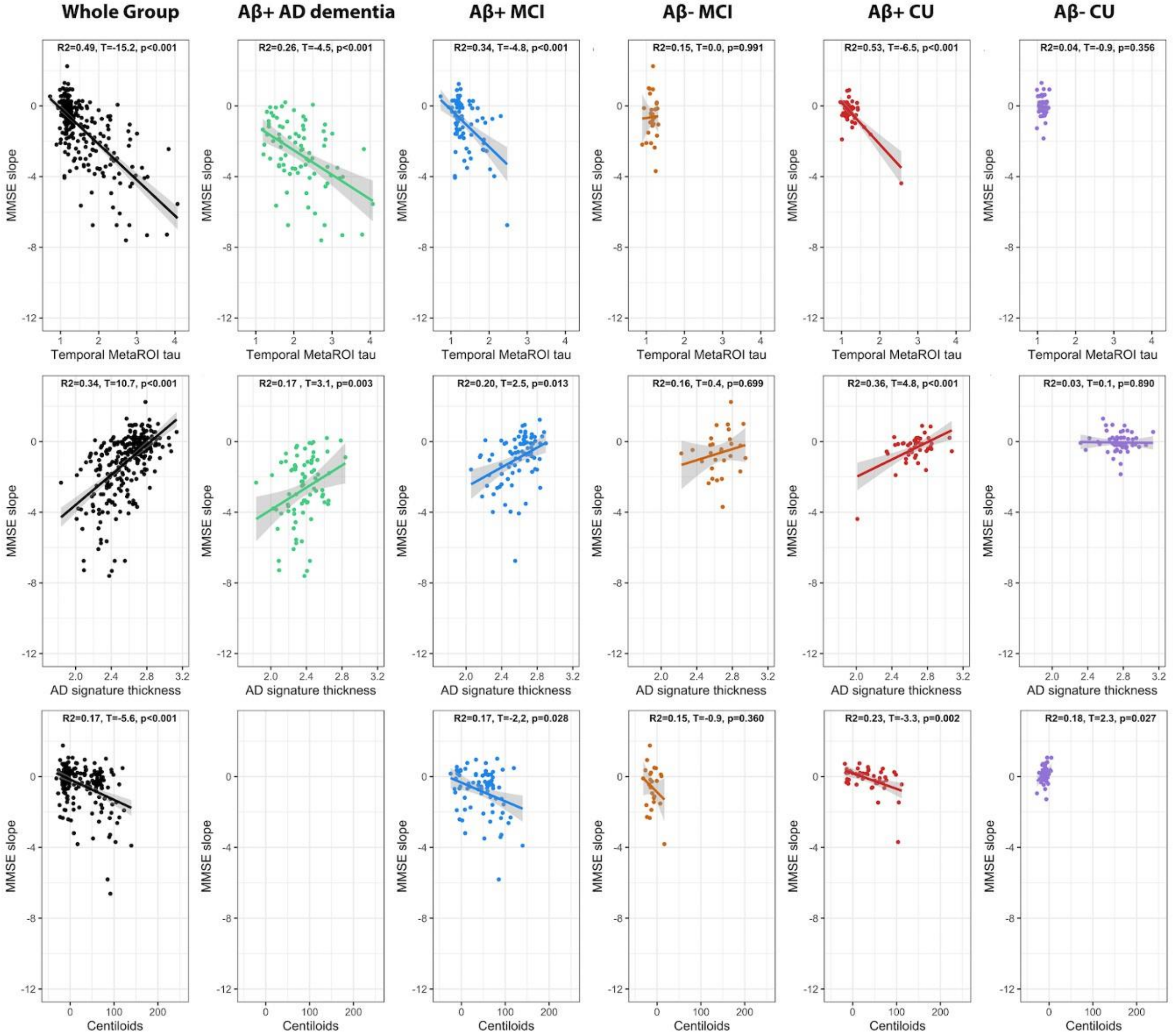


© 2021 Ossenkoppele R et al. *JAMA Neurology*.

Graphs represent associations between baseline [$^{18}$F]RO948 (tau) PET uptake in a temporal region-of-interest (upper panel), cortical thickness in an Alzheimer's disease signature region defined on MRI (middle panel) and Amyloid PET (bottom panel) with annual slopes of Mini-Mental State Examination scores across all participants (in black), Aβ+ Alzheimer's disease dementia (in green), Aβ+ mild cognitive impairment (in blue), Aβ- mild cognitive impairment (in orange), Aβ+ cognitively unimpaired individuals (in red) and Aβ- cognitively unimpaired individuals (in purple). Model outputs are derived from a linear regression model between baseline tau PET/MRI/amyloid PET and MMSE slopes, adjusted for age, sex, education and cohort. $R^2$ values are provided for the full model (including covariates), and T and p-values represent the interaction between the imaging modality and time. Note that amyloid-PET was only available in 5 participants with AD dementia of the Swedish BioFINDER-2 study and therefore now scatterplot was included for this group.

© 2021 Ossenkoppele R et al. *JAMA Neurology*.

eFigure 2. [$^{18}$F]Flortaucipir SUVR in Entorhinal Cortex and Hippocampal Volumes as Predictors of Change in MMSE

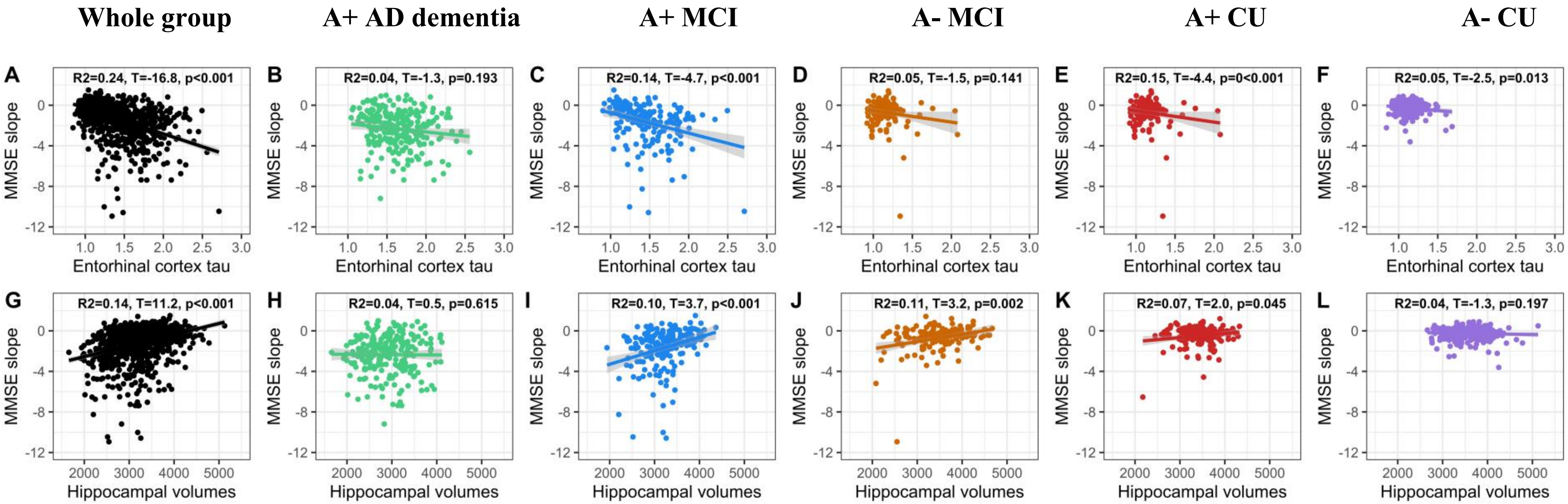


© 2021 Ossenkoppele R et al. *JAMA Neurology*.

eFigure 3. Replication of eFigure 2 With [$^{18}$F]RO948 PET in an Independent Dataset

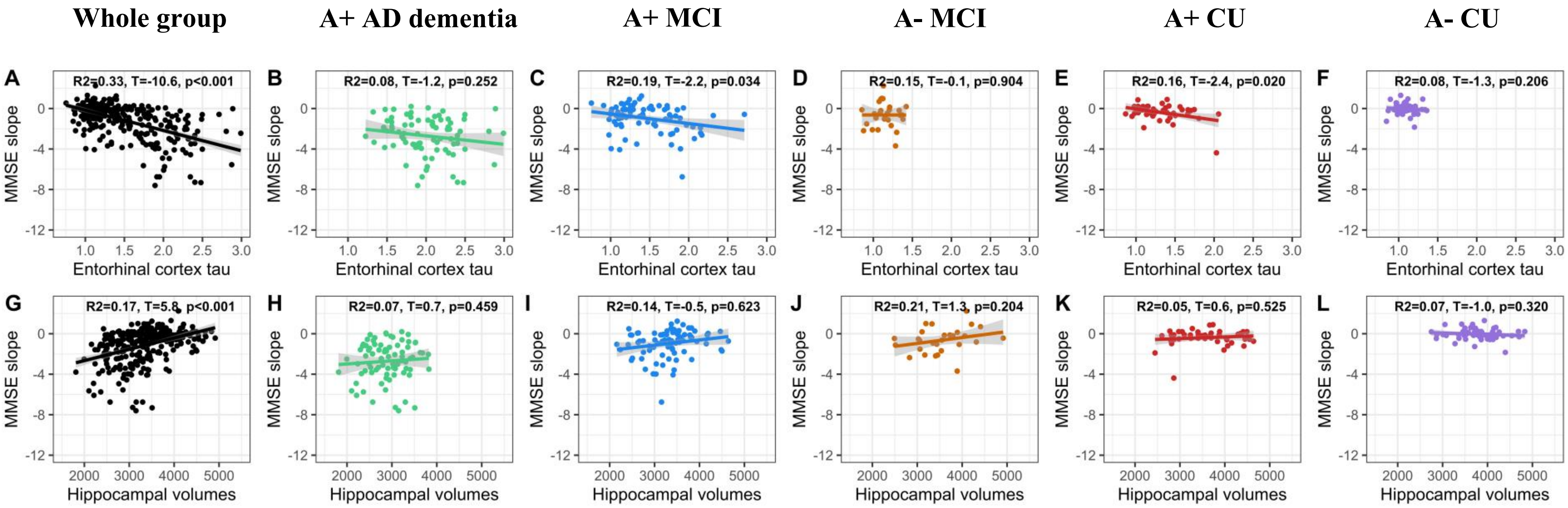


© 2021 Ossenkoppele R, et al. *JAMA Neurology*.

eFigure 4. [[18]F]Flortaucipir SUVR in Braak V/VI ROIs and Whole Brain Cortical Thickness as Predictors of Change in MMSE

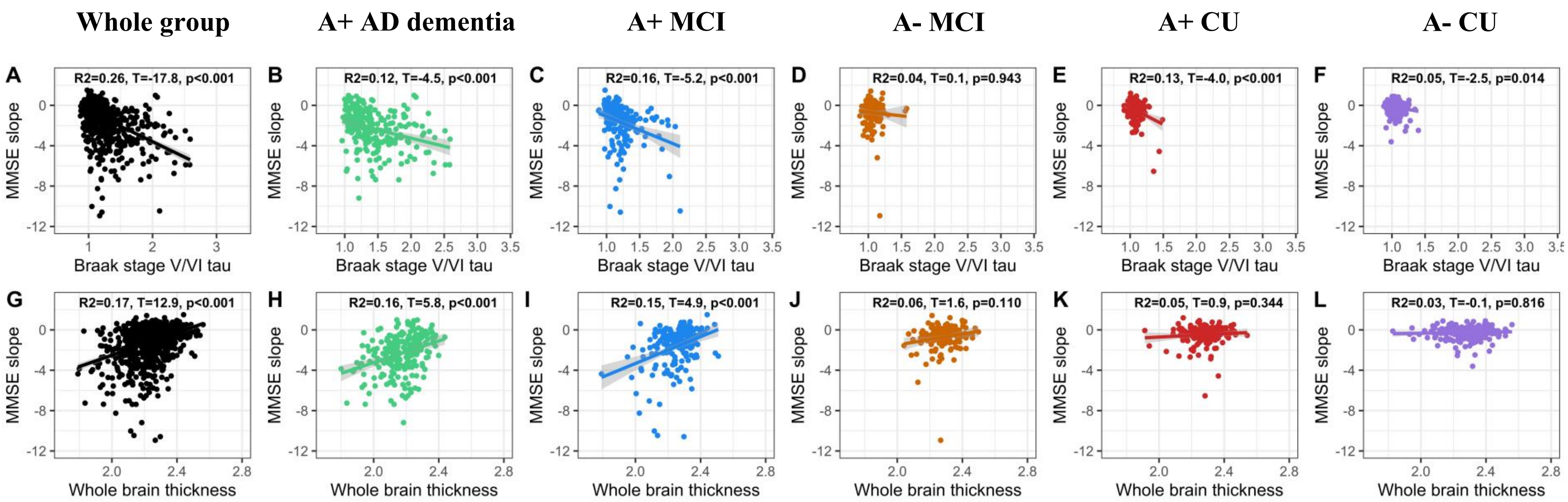


© 2021 Ossenkoppele R, et al. *JAMA Neurology*.

eFigure 5. Replication of eFigure 4 With [18F]RO948 PET in an Independent Dataset

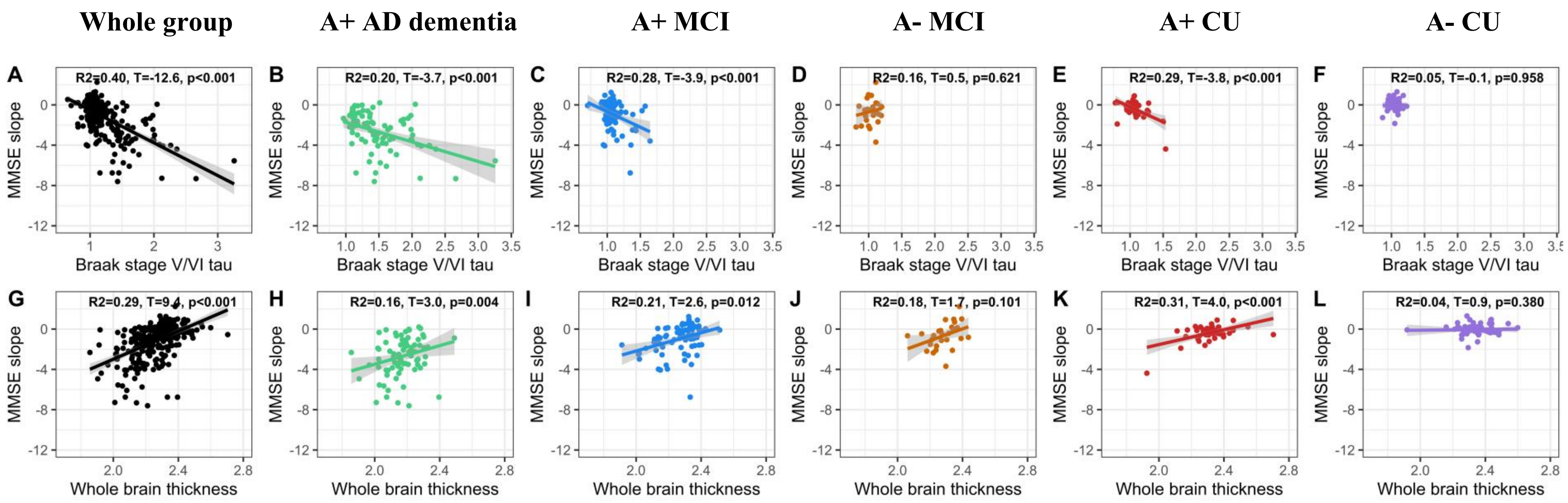


© 2021 Ossenkoppele R, et al. *JAMA Neurology*.

eFigure 6. Replication of Figure 2 (Mediation Analysis) Using [$^{18}$F]RO948 PET in an Independent Dataset

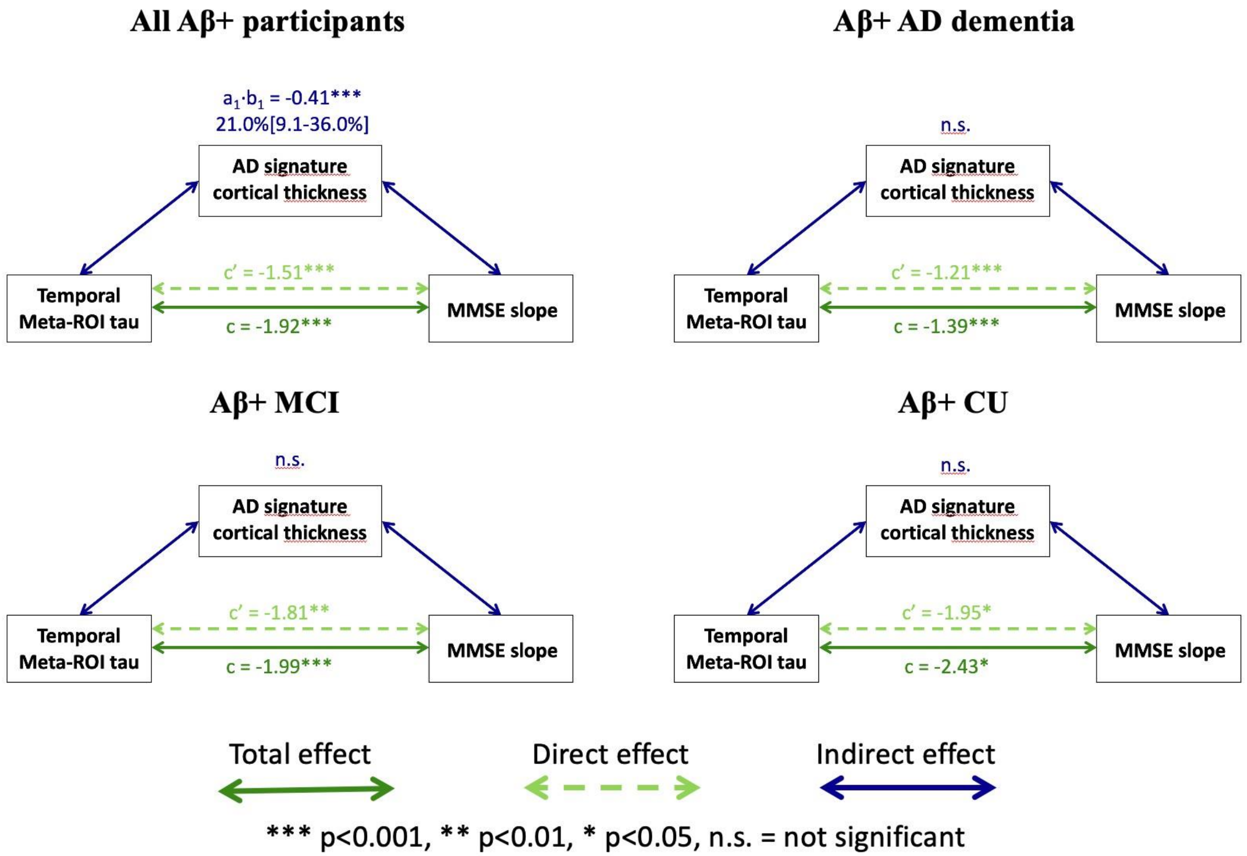


Path diagrams indicate whether AD-signature cortical thickness mediates the associations between baseline [$^{18}$F]RO948 SUVR in the temporal meta-ROI and MMSE slopes, adjusting for age, sex, education and *APOE* ε4 status. The direct effect (i.e., coefficient: c') reflects the extent to which MMSE slopes change when baseline tau PET increases by one unit while baseline cortical thickness remains unaltered. The indirect effect (i.e., coefficient: $a_1 \cdot b_1$) reflects the extent to which MMSE slopes changes when baseline tau PET is held constant and baseline cortical thickness changes by the amount it would have changed had baseline tau PET increased by one unit.The coefficient "c" represents the total effect (i.e., direct

© 2021 Ossenkoppele R, et al. *JAMA Neurology*.

+ indirect effects).

eFigure 7. Replication of Figure 3 (Associations With Age, Sex, and *APOE*) Using [$^{18}$F]RO948 PET in an Independent Dataset

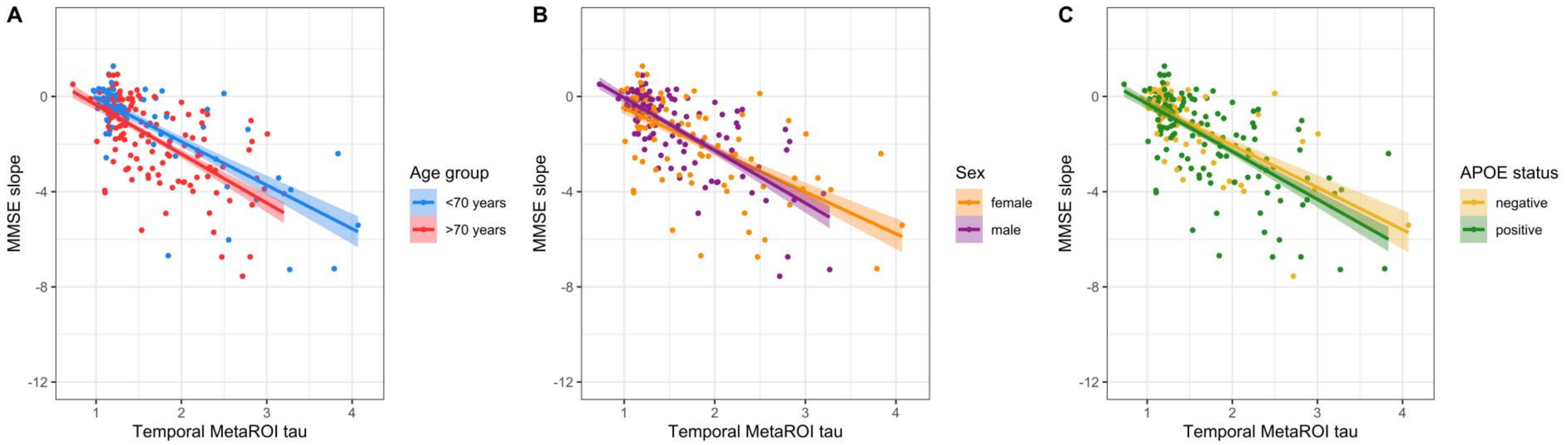


Linear mixed effects models with random intercepts and fixed slopes were performed to examine whether age (A), sex (B) and *APOE* ε4 status (C) moderate the association between baseline [$^{18}$F]RO948 uptake in a temporal region-of-interest and change over time in Mini-Mental State Examination (MMSE) scores, while adjusting for age, sex, education, cohort and diagnostic group when appropriate. The T- and p-value represent the 3-way interaction Age/Sex/*APOE* ε4 status * time * tau PET. Age was entered as continuous variable in the linear mixed effects models, but was dichotomized at age 70 for visualization purposes.

© 2021 Ossenkoppele R, et al. *JAMA Neurology*.

eTable 1. Participant Characteristics for the Discovery Cohort (A) vs the Replication Cohort (B)

A. [$^{18}$F]Flortaucipir

| | **Whole group** | **Aβ+ AD dementia** | **Aβ+ MCI** | **Aβ- MCI** | **Aβ+ CU** | **Aβ- CU** |
|---|---|---|---|---|---|---|
| N | 1135 | 235 | 190 | 144 | 208 | 358 |
| Age, years | 71.6 (8.3) | 71.7 (8.9) | 71.5 (8.2) | 70.4 (8.5) | 74.3 (6.3) | 70.5 (8.5) |
| Sex (% male) | 52.2 | 62.1 | 48.4 | 42.4 | 50.5 | 52.8 |
| Education, years | 13.7 (6.4) | 12.6 (5.1) | 12.1 (5.4) | 12.7 (5.9) | 16.1 (9.4) | 14.2 (5.2) |
| *APOE* ε4, % positive | 41.1% | 67.3% | 60.2% | 20.3% | 52.5% | 17.3% |
| MMSE, baseline score | 26.7 (3.7) | 21.6 (5.1) | 27.0 (2.6) | 28.1 (1.8) | 28.9 (1.2) | 29.0 (1.2) |

B. [$^{18}$F]RO948

| | **Whole group** | **Aβ+ AD dementia** | **Aβ+ MCI** | **Aβ- MCI** | **Aβ+ CU** | **Aβ- CU** |
|---|---|---|---|---|---|---|
| N | 296 | 80 | 81 | 28 | 45 | 62 |
| Age, years | 69.6 (10.3) | 74.0 (6.5) | 72.1 (7.1) | 68.9 (6.8) | 70.2 (9.8) | 60.9 (13.6) |
| Sex (% male) | 53.4 | 47.5 | 55.6 | 64.3 | 44.4 | 59.7 |
| Education, years | 12.6 (4.2) | 12.1 (4.7) | 12.5 (4.4) | 11.8 (2.7) | 13.1 (4.1) | 13.3 (3.5) |
| *APOE* ε4, % positive | 56.8% | 68.4% | 66.2% | 21.4% | 68.9% | 37.1% |
| MMSE, baseline score | 25.9 (4.6) | 19.9 (4.4) | 27.1 (1.9) | 27.3 (2.3) | 28.8 (1.2) | 29.1 (1.1) |

Data are presented as mean (standard deviation), unless otherwise stated.
Aβ = amyloid-β, AD = Alzheimer's disease, *APOE* = Apolipoprotein E, CU = Cognitively unimpaired, MCI = Mild cognitive impairment, MMSE = Mini-mental state examination.

© 2021 Ossenkoppele R, et al. *JAMA Neurology*.

eTable 2. Participant Characteristics for the Different Cohorts

BioFINDER-1:

| | Whole group | Aβ+ AD dementia | Aβ+ MCI | Aβ- MCI | Aβ+ CU | Aβ- CU |
|---|---|---|---|---|---|---|
| N | 136 | 44 | 27 | 1 | 32 | 32 |
| Age, years | 72.3 (10.3) | 71.0 (7.5) | 71.6 (9.5) | 66 | 74.1 (8.0) | 73.0 (7.1) |
| Sex (% male) | 54.4 | 56.8 | 66.7 | 100 | 37.5 | 56.2 |
| Education, years | 12.4 (3.7) | 12.3 (3.8) | 12.4 (3.7) | 13.9 | 11.9 (3.7) | 12.8 (3.8) |
| *APOE* ε4, % positive | 59.2% | 64.9% | 80.8% | 0% | 80.0% | 16.1% |
| MMSE, baseline score | 25.7 (4.7) | 20.8 (4.9) | 25.8 (2.9) | 30.0 | 28.9 (1.1) | 28.8 (1.1) |

Seoul:

| | Whole group | Aβ+ AD dementia | Aβ+ MCI | Aβ- MCI | Aβ+ CU | Aβ- CU |
|---|---|---|---|---|---|---|
| N | 161 | 42 | 37 | 16 | 8 | 58 |
| Age, years | 69.4 (9.6) | 72.7 (9.5) | 71.8 (8.6) | 69.8 (9.9) | 71.0 (4.4) | 65.2 (9.4) |
| Sex (% male) | 64.0 | 76.2 | 54.1 | 68.1 | 62.5 | 60.3 |
| Education, years | 11.5 (4.9) | 10.2 (5.8) | 11.8 (4.6) | 10.6 (4.4) | 12.5 (4.1) | 12.4 (4.4) |
| *APOE* ε4, % positive | 35.0% | 58.5% | 48.6% | 12.5% | 37.5% | 15.5% |
| MMSE, baseline score | 25.0 (5.0) | 19.0 (5.3) | 25.4 (3.2) | 26.5 (2.3) | 27.9 (2.1) | 28.3 (1.7) |

© 2021 Ossenkoppele R, et al. *JAMA Neurology*.

UCSF:

| | Whole group | Aβ+ AD dementia | Aβ+ MCI | Aβ- MCI | Aβ+ CU | Aβ- CU |
|---|---|---|---|---|---|---|
| N | 44 | 28 | 8 | 6 | 1 | 2 |
| Age, years | 63.9 (10.1) | 64.6 (10.4) | 61.8 (10.0) | 67.3 (7.2) | 70 | 60.9 (13.6) |
| Sex (% male) | 61.4 | 70.4 | 62.5 | 33.3 | 100 | 0 |
| Education, years | 16.9 (2.7) | 22.5 (3.9) | 17.9 (3.1) | 16.8 (1.3) | 20.0 | 18.5 (2.1) |
| *APOE* ε4, % positive | 45.7% | 52.5% | 40.0% | 0% | 0% | 100% |
| MMSE, baseline score | 24.6 (4.2) | 6.4 (2.7) | 17.9 (3.1) | 16.8 (1.3) | 27.0 | 30.0 (0) |

ADNI:

| | Whole group | Aβ+ AD dementia | Aβ+ MCI | Aβ- MCI | Aβ+ CU | Aβ- CU |
|---|---|---|---|---|---|---|
| N | 445 | 17 | 77 | 83 | 116 | 152 |
| Age, years | 71.6 (6.7) | 72.1 (10.1) | 72.0 (6.9) | 70.8 (7.8) | 73.5 (6.3) | 70.4 (5.5) |
| Sex (% male) | 49.0 | 35.3 | 39.0 | 33.7 | 57.8 | 57.2 |
| Education, years | 16.5 (2.6) | 16.4 (2.6) | 15.8 (2.6) | 16.5 (2.7) | 16.6 (2.4) | 16.7 (2.6) |
| *APOE* ε4, % positive | 36.2% | 76.9% | 62.7% | 17.5% | 47.4% | 20.1% |
| MMSE, baseline score | 28.5 (1.9) | 22.6 (2.6) | 27.7 (2.0) | 28.5 (1.4) | 29.0 (1.2) | 29.2 (1.0) |

© 2021 Ossenkoppele R, et al. *JAMA Neurology*.

AO5:

| | **Whole group** | **Aβ+ AD dementia** | **Aβ+ MCI** | **Aβ- MCI** | **Aβ+ CU** | **Aβ- CU** |
|---|---|---|---|---|---|---|
| N | 160 | 26 | 41 | 38 | 5 | 50 |
| Age, years | 71.3 (9.6) | 76.5 (8.2) | 72.2 (8.2) | 70.3 (9.7) | 77.8 (7.0) | 67.9 (10.3) |
| Sex (% male) | 48.1 | 57.5 | 46.3 | 50.0 | 40.0 | 44.0 |
| Education, years | 13.7 (6.4) | 12.6 (2.6) | 12.6 (2.4) | 13.7 (6.7) | 13.2 (3.4) | 15.2 (9.3) |
| *APOE* ε4, % positive | 41.4% | 73.1% | 55.3% | 33.3% | 40.0% | 19.1% |
| MMSE, baseline score | 27.4 (3.4) | 21.5 (4.1) | 27.5 (1.9) | 28.2 (1.8) | 29.6 (0.6) | 29.5(0.5) |

EXPEDITION-3:

| | **Whole group** | **Aβ+ AD dementia** | **Aβ+ MCI** | **Aβ- MCI** | **Aβ+ CU** | **Aβ- CU** |
|---|---|---|---|---|---|---|
| N | 79 | 79 | NA | NA | NA | NA |
| Age, years | 72.4 (7.5) | 72.4 (7.5) | NA | NA | NA | NA |
| Sex (% male) | 62.0 | 62.0 | NA | NA | NA | NA |
| Education, years | 14.7 (2.9) | 14.7 (2.9) | NA | NA | NA | NA |
| *APOE* ε4, % positive | 74.0% | 74.0% | NA | NA | NA | NA |
| MMSE, baseline score | 22.9 (2.0) | 22.9 (2.0) | NA | NA | NA | NA |

© 2021 Ossenkoppele R, et al. *JAMA Neurology*.

BACS:

| | Whole group | Aβ+ AD dementia | Aβ+ MCI | Aβ- MCI | Aβ+ CU | Aβ- CU |
|---|---|---|---|---|---|---|
| N | 110 | NA | NA | NA | 46 | 64 |
| Age, years | 77.1 (6.1) | NA | NA | NA | 77.0 (7.2) | 77.3 (7.2) |
| Sex (% male) | 40.9 | NA | NA | NA | 39.1 | 42.2 |
| Education, years | 18.2 (12.1) | NA | NA | NA | 19.4 (18.4) | 17.4 (2.8) |
| *APOE* ε4, % positive | 26.6% | NA | NA | NA | 51.1% | 9.4% |
| MMSE, baseline score | 28.7 (1.2) | NA | NA | NA | 28.6 (1.4) | 28.8 (1.1) |

BioFINDER-2:

| | Whole group | Aβ+ AD dementia | Aβ+ MCI | Aβ- MCI | Aβ+ CU | Aβ- CU |
|---|---|---|---|---|---|---|
| N | 296 | 80 | 81 | 28 | 45 | 62 |
| Age, years | 69.6 (10.3) | 74.0 (6.5) | 72.1 (7.1) | 68.9 (6.8) | 70.2 (9.8) | 60.9 (13.6) |
| Sex (% male) | 53.4 | 47.5 | 55.6 | 64.3 | 44.4 | 59.7 |
| Education, years | 12.6 (4.2) | 12.1 (4.7) | 12.5 (4.4) | 11.8 (2.7) | 13.1 (4.1) | 13.3 (3.5) |
| *APOE* ε4, % positive | 56.8% | 68.4% | 66.2% | 21.4% | 68.9% | 37.1% |
| MMSE, baseline score | 25.9 (4.6) | 19.9 (4.4) | 27.1 (1.9) | 27.3 (2.3) | 28.8 (1.2) | 29.1 (1.1) |

Data are presented as mean (standard deviation), unless otherwise stated.
Aβ = amyloid-β, AD = Alzheimer's disease, *APOE* = Apolipoprotein E, CU = Cognitively unimpaired, MCI = Mild cognitive impairment, MMSE = Mini-mental state examination.

© 2021 Ossenkoppele R, et al. *JAMA Neurology*.

eTable 3. Bootstrapping of $R^2$ Values From Tau PET vs MRI Models Predicting MMSE Change

| | DISCOVERY COHORT ([$^{18}$F]flortaucipir) | | | | REPLICATION COHORT ([$^{18}$F]RO948) | | | |
|---|---|---|---|---|---|---|---|---|
| | $R^2$ difference PET - MRI | 95% CI | T | P | $R^2$ difference PET - MRI | 95% CI | T | P |
| **Total group** | 0.099 | 0.970-0.101 | 80.3 | **<0.001** | 0.137 | 0.136-0.139 | 147.9 | **<0.001** |
| **Aβ+ AD dementia** | -0.041 | -[0.045-0.036] | -17.2 | **<0.001** | 0.134 | 0.129-1.140 | 50.6 | **<0.001** |
| **Aβ+ MCI** | 0.104 | 0.097-0.110 | 30.8 | **<0.001** | 0.086 | 0.079-0.094 | 23.1 | **<0.001** |
| **Aβ+ CU** | 0.098 | 0.093-0.103 | 38.6 | **<0.001** | 0.184 | 0.163-0.206 | 16.7 | **<0.001** |
| **Aβ- MCI** | -0.092 | -[0.094-0.091] | -114.0 | **<0.001** | -0.047 | -[0.048-0.045] | -61.4 | **<0.001** |
| **Aβ- CU** | 0.022 | 0.018-0.025 | 13.6 | **<0.001** | 0.010 | 0.008-0.011 | 15.4 | **<0.001** |

© 2021 Ossenkoppele R, et al. *JAMA Neurology*.

eTable 4. Linear Mixed Models With [$^{18}$F]RO948 PET and MRI as Predictors of Change in MMSE

| | β (SE) | P | $R^2$ | AIC | $X^2$ for difference | P for difference |
|---|---|---|---|---|---|---|
| **ALL Aβ+ PARTICIPANTS** | | | | | | |
| Model 1: Age, sex, education, cohort | | | 0.066 | 3070 | | |
| Model 2: Model 1 + Tau PET | -0.22 (0.02) | **<0.001** | 0.596 | 2775 | 307.3 | **<0.001** |
| Model 3: Model 1 + Tau PET + MRI | -0.22 (0.02) | **<0.001** | 0.729 | 2633 | 107.8 | **<0.001** |
| | | | | | | |
| Model 2: Model 1 + MRI | 0.49 (0.05) | **<0.001** | 0.409 | 2868 | 176.2 | **<0.001** |
| Model 3: Model 1 + MRI + Tau PET | 0.48 (0.05) | **<0.001** | 0.723 | 2666 | 205.5 | **<0.001** |
| **Aβ+ AD DEMENTIA** | | | | | | |
| Model 1: Age, sex, education, cohort | | | 0.152 | 1187 | | |
| Model 2: Model 1 + Tau PET | -0.15 (0.04) | **<0.001** | 0.384 | 1142 | 46.8 | **<0.001** |
| Model 3: Model 1 + Tau PET + MRI | -0.15 (0.04) | **<0.001** | 0.419 | 1137 | 6.6 | **<0.001** |
| | | | | | | |
| Model 2: Model 1 + MRI | 0.42 (0.14) | **0.002** | 0.282 | 2265 | 24.0 | **<0.001** |
| Model 3: Model 1 + MRI + Tau PET | 0.42 (0.14) | **0.002** | 0.413 | 1144 | 22.9 | **<0.001** |
| **Aβ+ MILD COGNITIVE IMPAIRMENT** | | | | | | |
| Model 1: Age, sex, education, cohort | | | 0.125 | 1017 | | |
| Model 2: Model 1 + Tau PET | -0.27 (0.05) | **<0.001** | 0.300 | 973 | 48.4 | **<0.001** |
| Model 3: Model 1 + Tau PET + MRI | -0.27 (0.05 | **<0.001** | 0.308 | 971 | 1.6 | **<0.001** |
| | | | | | | |
| Model 2: Model 1 + MRI | 0.35 (0.09) | **<0.001** | 0.210 | 999 | 22.9 | **<0.001** |
| Model 3: Model 1 + MRI + Tau PET | 0.35 (0.09) | **<0.001** | 0.274 | 988 | 11.2 | **<0.001** |
| **Aβ+ COGNITIVELY UNIMPAIRED INDIVIDUALS** | | | | | | |
| Model 1: Age, sex, education, cohort | | | 0.096 | 418 | | |
| Model 2: Model 1 + Tau PET | -0.25 (0.06) | **<0.001** | 0.382 | 381 | 40.3 | **<0.001** |
| Model 3: Model 1 + Tau PET + MRI | -0.25 (0.06) | **<0.001** | 0.384 | 381 | 1.0 | 0.311 |
| | | | | | | |
| Model 2: Model 1 + MRI | 0.13 (0.08) | 0.098 | 0.161 | 414 | 7.6 | **0.023** |
| Model 3: Model 1 + MRI + Tau PET | 0.16 (0.08) | **0.038** | 0.318 | 395 | 21.3 | **<0.001** |

© 2021 Ossenkoppele R, et al. *JAMA Neurology*.

eTable 5. Bootstrapping of $R^2$ Values From Tau PET vs Amyloid PET Models Predicting MMSE Change

| | DISCOVERY COHORT ([18F]flortaucipir) | | | | REPLICATION COHORT ([18F]RO948) | | | |
|---|---|---|---|---|---|---|---|---|
| | $R^2$ difference TAU – Aβ | 95% CI | T | P | $R^2$ difference TAU – Aβ | 95% CI | T | P |
| **Total group** | 0.181 | 0.178-0.183 | 147.1 | **<0.001** | 0.139 | 0.135-0.143 | 64.5 | **<0.001** |
| **Aβ+ AD dementia** | 0.149 | 0.146-0.153 | 81.1 | **<0.001** | NA | NA | NA | **NA** |
| **Aβ+ MCI** | 0.190 | 0.184-0.196 | 63.3 | **<0.001** | 0.192 | 0.184-0.199 | 49.8 | **<0.001** |
| **Aβ+ CU** | 0.102 | 0.098-0.106 | 47.0 | **<0.001** | 0.259 | 0.240-0.279 | 26.3 | **<0.001** |
| **Aβ- MCI** | 0.002 | 0.002-0.003 | 8.1 | **<0.001** | -0.065 | -[0.067-0.063] | -61.5 | **<0.001** |
| **Aβ- CU** | 0.034 | 0.031-0.037 | 21.7 | **<0.001** | -0.096 | -[0.099-0.093] | -63.2 | **<0.001** |

© 2021 Ossenkoppele R, et al. *JAMA Neurology*.

eTable 6. Linear Mixed Models With [18F]Flortaucipir PET and Amyloid PET as Predictors of Change in MMSE

| | β (SE) | P | $R^2$ | AIC | $X^2$ for difference | P for difference |
|---|---|---|---|---|---|---|
| **ALL Aβ+ PARTICIPANTS** | | | | | | |
| Model 1: Age, sex, education, cohort | | | 0.185 | 8333 | | |
| Model 2: Model 1 + Tau PET | -0.21 (0.02) | **<0.001** | 0.488 | 7867 | 470.0 | **<0.001** |
| Model 3: Model 1 + Tau PET + Amyloid PET | -0.21 (0.02) | **<0.001** | 0.492 | 7862 | 7.1 | **0.008** |
| | | | | | | |
| Model 2: Model 1 + Amyloid PET | 0.001 (0.0002) | **<0.001** | 0.254 | 8254 | 83.7 | **<0.001** |
| Model 3: Model 1 + Amyloid PET + Tau PET | 0.001 (0.0002) | **<0.001** | 0.463 | 8001 | 254.7 | **<0.001** |
| **Aβ+ AD DEMENTIA** | | | | | | |
| Model 1: Age, sex, education, cohort | | | 0.196 | 3129 | | |
| Model 2: Model 1 + Tau PET | -0.18 (0.03) | **<0.001** | 0.329 | 3050 | 46.8 | **<0.001** |
| Model 3: Model 1 + Tau PET + Amyloid PET | -0.18 (0.03) | **<0.001** | 0.329 | 3052 | 0.1 | 0.822 |
| | | | | | | |
| Model 2: Model 1 + Amyloid PET | -0.0003 (0.0004) | 0.386 | 0.198 | 3131 | 1.3 | 0.522 |
| Model 3: Model 1 + Amyloid PET + Tau PET | -0.0004 (0.0004) | 0.328 | 0.302 | 3091 | 41.8 | **<0.001** |
| **Aβ+ MILD COGNITIVE IMPAIRMENT** | | | | | | |
| Model 1: Age, sex, education, cohort | | | 0.225 | 2855 | | |
| Model 2: Model 1 + Tau PET | -0.25 (0.03) | **<0.001** | 0.387 | 2755 | 104.5 | **<0.001** |
| Model 3: Model 1 + Tau PET + Amyloid PET | -0.25 (0.03) | **<0.001** | 0.386 | 2757 | 0.0 | 0.972 |
| | | | | | | |
| Model 2: Model 1 + Amyloid PET | -0.0007 (0.0003) | **0.039** | 0.237 | 2852 | 7.8 | **0.020** |
| Model 3: Model 1 + Amyloid PET + Tau PET | -0.0007 (0.0003) | **0.032** | 0.338 | 2807 | 46.3 | **<0.001** |
| **Aβ+ COGNITIVELY UNIMPAIRED INDIVIDUALS** | | | | | | |
| Model 1: Age, sex, education, cohort | | | 0.095 | 1680 | | |
| Model 2: Model 1 + Tau PET | -0.17 (0.06) | **0.004** | 0.175 | 1658 | 25.9 | **<0.001** |
| Model 3: Model 1 + Tau PET + Amyloid PET | -0.17 (0.06) | **0.004** | 0.175 | 1659 | 0.2 | 0.694 |
| | | | | | | |
| Model 2: Model 1 + Amyloid PET | -0.0005 (0.0002) | **0.023** | 0.114 | 1675 | 8.4 | **0.015** |
| Model 3: Model 1 + Amyloid PET + Tau PET | -0.0005 (0.0002) | **0.023** | 0.168 | 1662 | 14.7 | **<0.001** |

© 2021 Ossenkoppele R, et al. *JAMA Neurology*.

**eAppendix.** ADNI Investigators

Michael Weiner
Paul Aisen
Ronald Petersen
Clifford R. Jack Jr.
William Jagust
John Q. Trojanowski
Arthur W. Toga
Laurel Beckett
Robert C. Green
Andrew J. Saykin
John Morris
Leslie M. Shaw
Enchi Liu
Tom Montine
Ronald G. Thomas
Michael Donahue
Sarah Walter
Devon Gessert
Tamie Sather
Gus Jiminez
Danielle Harvey
Natthew Bernstein
Nick Fox
Paul Thompson
Norbert Schuff
Charles DeCarli
Bret Borowski
Jeff Gunter
Matt Senjem
Prashanti Vemuri
David Jones
Kejal Kantarci
Chard Ward
Robert A. Koeppe

© 2021 Ossenkoppele R, et al. *JAMA Neurology*.

Norm Foster
Eric M. Reiman
Kewei Chen
Chet Mathis
Susan Landau
Nigel J. Cairns
Erin Householder
Lisa T. Reinwald
Virginia Lee
Magdalena Korecka
Michal Figurski
Karen Crawford
Scott Neu
Tatiana M. Foroud
Steven Potkin
Li Chen
Faber Kelley
Sungeun Kim
Kwangsik Nho
Zaven Kachaturian
Richard Frank
Peter J. Snyder
Susan Molchan
Jeffrey Kaye
Joseph Quinn
Betty Lind
Raina Carter
Sara Dolen
Lon S. Schneider
Sonia Pawluczyk
Mauricio Beccera
Liberty Teodoro
Bryan M. Spann
James Brewer
Helen Vanderswag
Adam Fleisher

© 2021 Ossenkoppele R, et al. *JAMA Neurology*.

Judith L. Heidebrink
Joanne L. Ford
Sara S. Mason
Colleen S. Albers
David Knopman
Kris Johnson
Rachelle S. Doody
Javier Villanueva
Munir Chowdhury
Susan Rountree
Mimi Dang
Yaakov Stern
Lawrence S. Honig
Karen L. Bell
Beau Ances
Maria Carroll
Sue Leon
Erin Householder
Mark A. Mintun
Stacy Schneider
Angela Oliver
Randal Griffith
David Clark
David Geldmacher
John Brockington
Erik Roberson
Hillel Grossman
Effie Mitsis
Leyla deToledo-Morrell
Raj C. Shah
Ranjan Duara
Daniel Varon
Maria T. Greig
Peggy Roberts
Marilyn Albert
Chiadi Onyike

© 2021 Ossenkoppele R, et al. *JAMA Neurology*.

Daniel D'Agostino
Stephanie Kleib
James E. Galvin
Dana M. Pogorelec
Brittany Cerbone
Christina A. Michel
Henry Rusinek
Mony J. De Leon
Lidia Glodzik
Susan De Santi
Murali Doraiswamy
Jeffrey R. Petrella
Terence Z. Wong
Jason H. Karlawish
David Wolk
Charles D. Smith
Greg Jicha
Peter Hardy
Partha Sinha
Elizabeth Oates
Gary Conrad
Oscar L. Lopez
MaryAnn Oakley
Donna M. Simpson
Anton P. Porsteinsson
Bonnie S. Goldstein
Kim Martin
Kelly M. Makino
M. Saleem Ismael
Connie Brand
Ruth A. Mulnard
Gaby Thai
Catherina McAdams Ortiz
Kyle Womack
Dana M. Mathews
Mary Quiceno

© 2021 Ossenkoppele R, et al. *JAMA Neurology*.

Ramon Diaz Arrastia
Richard King
Myron Steiner
Kristen Martin Cook
Michael Devous
Allan I. Levey
James J. Lah
Janet S. Cellar
Jeffrey M. Burns
Heather S. Anderson
Russell h. Swerdlov
Liana Apostolova
Kathleen Tingus
Ellen Woo
Daniel H. Silverman
Po H. Lu
George Bartzokis
Neill R. Graff-Radford
Francine Parfitt
Tracey Kendall
Heather S. Johnson
Martin R. Farlow
Ann-Marie Hake
Brandy R. Matthews
Scott Herring
Cynthia Hunt
Christopher H. van Dyck
Richard E. Carson
Martyha G. MacAvoy
Howard Chertkow
Howard Bergman
Chris Hosein
Sandra Black
Bojana Stefanovic
Curtis Caldwell
Ging Yuek Robin

© 2021 Ossenkoppele R, et al. *JAMA Neurology*.

Howard Feldman
Benita Mudge
Michele Assaly Past
Andrew Kertesz
John Rogers
Dick Trost
Charles Bernick
Donna Munic
Diana Kerwin
Marek M. Mesulam
Kristine Lipowski
Chuang Kuo Wu
Nancy Johnson
Carl Sadowsky
Walter Martinez
Raymond S. Turner
Kathleen Johnson
Brigid Reynolds
Reisa A. Sperling
Keith A. Johnson
Gad Marshall
Meghan Frey
Jerome Yesavage
Joy L. Taylor
Barton Lane
Allyson Rosen
Jared Tinklenberg
Marwan N. Sabbagh
Christine M. Belden
Sherye A. Sirrel
Neil Kowal
Ronald Killiany
Andrew E. Budson
Alexander Norbash
Patricia L. Johnson
Thomas O. Obisesan

© 2021 Ossenkoppele R, et al. *JAMA Neurology*.

Saba Wolday
Joana Allard
Alan Lerner
Paula Ogrocki
Leon Hudson
Evan Fletcher
Owen Carmichel
John Olichney
Smita Kittur
Michael Borrie
T.Y. Lee
Rob Bartha
Sterling Johnson
Sanjay Asthana
Cynthia M. Carlsson
Steven G. Potkin
Adrian Preda
Daan Nguyen
Pierre Tarlot
Stephanie Reeder
Vernice Bates
Horacio Capote
Michelle Rainka
Douglas W. Scharre
Maria Kataki
Anahita Adeli
Earl A. Zimmerman
Dzintra Celmins
Alice D. Brown
Godfrey D. Pearlson
Karen Blank
Karen Anderson
Robert B. Santulli
Tamar J. Kitzmiller
Eben S, Schwartz
Kaycee M. Sinks

© 2021 Ossenkoppele R, et al. *JAMA Neurology*.

Jeff D. Williamson
Pradeep Garg
Franklin Watkins
Brian R. Ott
Henry Querfurth
Geoffry Tremont
Stephen Salloway
Paul Malloy
Stephen Correia
Howard J. Rosen
Bruce L. Miller
Jacob Mintzer
Kenneth Spicer
David Bachman
Elizabether Pasternak
Irina Rachiksky
John Rogers
Andrew Kertesz
Nunzio Pomara
Raymundo Hernando
Antero Sarrael
Susan K. Schultz
Laura L. Boles
Hyungsub Shim
Karen E. Smith
Norman Relkin
Gloria Chaing
Amanda Smith
Kristin. Fargher
Balebail A. Raj

© 2021 Ossenkoppele R, et al. *JAMA Neurology*.
